# Anaerobic benzene oxidation in *Geotalea daltonii* involves activation by methylation and is regulated by the transition state regulator AbrB

**DOI:** 10.1128/aem.00856-24

**Published:** 2024-09-17

**Authors:** James E. Bullows, Alison Kanak, Lawrence Shedrick, Christina Kiessling, Muktak Aklujkar, Joel Kostka, Kuk-Jeong Chin

**Affiliations:** 1 Department of Biology, Georgia State University, Atlanta, Georgia, USA; 2 Department of Microbiology, University of Massachusetts, Amherst, Massachusetts, USA; 3 School of Earth and Atmospheric Sciences, Georgia Institute of Technology, Atlanta, Georgia, USA; 4 School of Biological Sciences, Georgia Institute of Technology, Atlanta, Georgia, USA; 5 Center for Microbial Dynamics and Infection, Georgia Institute of Technology, Atlanta, Georgia, USA; Danmarks Tekniske Universitet The Novo Nordisk Foundation Center for Biosustainability, Kgs. Lyngby, Denmark

**Keywords:** *Geotalea daltonii*, benzene, toluene, methylation, Bss, Hbs, AbrB, anaerobic aromatic degradation

## Abstract

Benzene is a widespread groundwater contaminant that persists under anoxic conditions. The aim of this study was to more accurately investigate anaerobic microbial degradation pathways to predict benzene fate and transport. Preliminary genomic analysis of *Geotalea daltonii* strain FRC-32, isolated from contaminated groundwater, revealed the presence of putative aromatic-degrading genes. *G. daltonii* was subsequently shown to conserve energy for growth on benzene as the sole electron donor and fumarate or nitrate as the electron acceptor. The *hbs* gene, encoding for 3-hydroxybenzylsuccinate synthase (Hbs), a homolog of the radical-forming, toluene-activating benzylsuccinate synthase (Bss), was upregulated during benzene oxidation in *G. daltonii*, while the *bss* gene was upregulated during toluene oxidation. Addition of benzene to the *G. daltonii* whole-cell lysate resulted in toluene formation, indicating that methylation of benzene was occurring. Complementation of σ^54^*-* (deficient) *E. coli* transformed with the *bss* operon restored its ability to grow in the presence of toluene, revealing *bss* to be regulated by σ^54^. Binding sites for σ^70^ and the transition state regulator AbrB were identified in the promoter region of the σ^54^-encoding gene *rpoN,* and binding was confirmed. Induced expression of *abrB* during benzene and toluene degradation caused *G. daltonii* cultures to transition to the death phase. Our results suggested that *G. daltonii* can anaerobically oxidize benzene by methylation, which is regulated by σ^54^ and AbrB. Our findings further indicated that the benzene, toluene, and benzoate degradation pathways converge into a single metabolic pathway, representing a uniquely efficient approach to anaerobic aromatic degradation in *G. daltonii*.

**IMPORTANCE:**

The contamination of anaerobic subsurface environments including groundwater with toxic aromatic hydrocarbons, specifically benzene, toluene, ethylbenzene, and xylene, has become a global issue. Subsurface groundwater is largely anoxic, and further study is needed to understand the natural attenuation of these compounds. This study elucidated a metabolic pathway utilized by the bacterium *Geotalea daltonii* capable of anaerobically degrading the recalcitrant molecule benzene using a unique activation mechanism involving methylation. The identification of aromatic-degrading genes and AbrB as a regulator of the anaerobic benzene and toluene degradation pathways provides insights into the mechanisms employed by *G. daltonii* to modulate metabolic pathways as necessary to thrive in anoxic contaminated groundwater. Our findings contribute to the understanding of novel anaerobic benzene degradation pathways that could potentially be harnessed to develop improved strategies for bioremediation of groundwater contaminants.

## INTRODUCTION

Benzene, toluene, ethylbenzene, and xylene (BTEX) are among the most frequently produced chemicals that represent widespread groundwater contaminants worldwide. BTEX compounds are well-known risks to public health; contamination has contributed to pregnancy complications, exposure has been linked to decreased immunity, and studies have determined them as carcinogens (1–3). Contaminated groundwater and aquifers are often anoxic, and remediation through oxygen injection has been shown to be effective but costly. Existing remediation methods have limitations (4), and further study is needed to develop bioremediation approaches such as bioaugmentation, involving the introduction of microbes with the ability to degrade recalcitrant compounds at contaminated environments (5). Anaerobic metabolism of BTEX has been reported in a number of microorganisms, including members of *Geobacteraceae* (6, 7), *Dechloromonas* (8), *Ferroglobus* (9), *Thauera* (10), *Azoarcus* (11), strain NaphS2 (12), strain N49 (13), other bacterial strains- NAP-3–1, NAP-3–2, and NAP-4 (14), mixed cultures PheN9 and TRIP1 (15, 16), and a benzene-degrading nitrate-reducing microbial consortium (17).

Anaerobic oxidation of aromatic hydrocarbons presents a unique challenge, especially unsubstituted aromatics such as benzene, due to the high dissociation energy necessary for C-C bond cleavage (18). The degradation of a stable benzene ring requires an electron density increase within the ring, referred to as activation (19). Two benzene-activating mechanisms have previously been reported: conversion to benzoate via carboxylation in the archaeon *Ferroglobus placidus* (9, 17) and to phenol via hydroxylation in *Geobacter metallireducens* (6, 20). Coates *et al*. (21) suggested methylation of benzene to toluene as a potential activation mechanism, analogous to the methylation of naphthalene in sulfate-reducing enrichment cultures (22, 23); this would represent an energetically more efficient mode of attack in benzene degradation that has heretofore not been documented (24–26). The putative methylation pathway is proposed to follow the anaerobic toluene degradation pathway after benzene is methylated to toluene (24). Toluene is converted to benzylsuccinate via the glycyl-radical utilizing enzyme benzylsuccinate synthase (Bss), encoded by the *bss* genes (27). The *bss* operon is composed of four genes, *bssA*, *bssB*, *bssC*, and *bssD* (28).

Anaerobic aromatic degradation pathways are regulated by proteins belonging to diverse families (29). For example, the *bam* genes, used during anaerobic toluene degradation, are regulated by an alternative sigma factor, σ^54^, particularly in the family *Geobacteraceae* (30, 31). Several studies reported that sigma factors are often modulated by transition state regulators (TSR) like AbrB, encoded by the *abrB* gene (32, 33). TSRs negatively regulate genes expressed in the stationary phase (34). Although the specific mechanism by which AbrB inhibits transcription is not fully understood, it has been reported that transcription is directly repressed by AbrB during exponential growth (35). AbrB has been shown as a transition state regulator in the anaerobic, aromatic-degrading *Bacillus subtilis* (36).

Preliminary genome analysis of *Geotalea daltonii* strain FRC-32 revealed the presence of putative aromatic-degrading genes and homologs, indicating its metabolic capacity for anaerobic aromatic hydrocarbon oxidation (37–39). The ability of *G. daltonii*’s close relative *G. metallireducens* to degrade toluene and benzene further indicates its potential to degrade aromatic hydrocarbons anaerobically (20, 38). *G. daltonii* was isolated from the radionuclides and hydrocarbon-contaminated subsurface of the U.S. Department of Energy Oak Ridge Field Research Center, Oak Ridge, Tennessee (40). This bacterium has adapted to the dynamic and oligotrophic subsurface by utilizing a wide range of terminal electron acceptors (ferric citrate, elemental sulfur, nitrate, malate, and fumarate) and electron donors (H_2_, formate, lactate, and butyrate) (40). Given its metabolic versatility, we investigated *G. daltonii* as an effective model pure culture system for understanding and manipulating the microbial metabolism of persistent aromatic hydrocarbons under anoxic conditions.

The aim of this study was to unravel the metabolic capacity of *G. daltonii* to oxidize benzene and other aromatic hydrocarbons anaerobically and elucidate the metabolic pathway of benzene oxidation. We demonstrated that *G. daltonii* indeed conserves energy for growth, with benzene as the sole electron donor and fumarate or nitrate as the electron acceptor. By closely coupling physiological analysis with molecular biology techniques, we revealed that benzene oxidation is activated by methylation using a unique mechanism and is regulated by the transition state regulator AbrB.

## MATERIALS AND METHODS

### Culturing conditions

*G. daltonii* strain FRC-32 (40) was grown anaerobically at 30°C in the dark in bicarbonate-buffered freshwater minimal medium (41) with the following modifications: 1.0 g NaCl, 0.4 g MgCl_2_ × 6 H_2_O, and 0.1 g CaCl_2_ × 2 H_2_O, and 0.2 g of cysteine hydrochloride, which was prepared under an atmosphere of N_2_/CO_2_ (80:20 [vol:vol]) in serum bottles sealed with thick butyl rubber septum stoppers (20 mm (D) x 20 mm (t)) and crimp seal as previously described (42, 43). The stoppers were used as the most robust stoppers available to account for recurrent needle penetrations for culture sampling to ensure that strictly anaerobic conditions were maintained for *G. daltonii* growth. Abiotic controls were carefully incubated to account for absorption of aromatic compounds within stoppers parallel to all experiments that were performed. Cultures were incubated at an angle to prevent contact with stoppers. *G. daltonii* cultures were grown on one of the following electron donors: acetate (5 mM), benzene (1 mM), toluene (1 mM), or benzoate (1 mM), and either fumarate (10 mM) or nitrate (1 mM) as an electron acceptor.

### Total RNA extraction

Total RNA was extracted from *G. daltonii* cultures at the mid-log growth phase as previously described (44) with some modifications, including addition of TM buffer [50 mM Tris-HCl (pH 7.0), 20 mM MgCl_2_ in DEPC-treated water] instead of TPM buffer, cell disruption for 1 minute at 2,500 rpm with a bead beater, and without yeast tRNA and RNase inhibitor. The isolated RNA was treated with TURBO DNase (Life Technologies, Grand Island, NY). RNA concentration and purity were determined with a Biophotometer (Eppendorf, Hamburg, Germany) and NanoDrop 2000 UV–Vis spectrophotometer (Thermo Fisher Scientific, Wilmington, DE).

### Primer design

The primers used in this study are listed in Table S1 and were synthesized by Integrated DNA Technologies (IDT) (San Jose, CA). All primers were designed specifically for this study based on the full genome sequence of *G. daltonii* using the PrimerQuest tool (45) and manually screened according to the primer design rules (46). Primers were tested for primer dimer and hairpin formation, and validity was confirmed via the IDT OligoAnalyzer tool (45).

### Reverse transcription-PCR (RT-PCR)

All cDNA syntheses were performed with gene-specific reverse primers, 0.5 µg total RNA, dNTP mix, RiboLock RNase inhibitor, and RevertAid RT reverse transcriptase (Thermo Fisher Scientific, Waltham, MA) incubated at 42°C for 60 minutes followed by enzyme inactivation at 70°C for 10 minutes. cDNA products were verified by PCR amplification via corresponding primer sets and visualized via gel electrophoresis.

### Quantitative reverse transcription-PCR (qRT-PCR)

Dilution series of purified RT-PCR amplicons obtained with gene-specific primers were used as calibration standards as described previously (44). All reactions were performed using SYBR Green PCR Master Mix (Life Technologies-Applied Biosystems, Grand Island, NY) and 20 pmol of each primer pair. The temperature profile was composed of an initial activation step at 50°C for 5 minutes and denaturation at 98°C for 40 seconds, followed by 40 cycles of denaturation at 98°C for 40 seconds, annealing at primer-specific temperature for 32 seconds, and elongation at 65°C for 32 seconds. Quantitative analysis was performed by the Applied Biosystems 7500 Real-Time PCR system (Life Technologies, Carlsbad, CA) with 7500 Real-Time PCR System Sequence Detection Software (Version 2.0.6). The precision and the reproducibility of quantification were carefully optimized. PCR product size and specificity were confirmed via agarose gel electrophoresis and Sanger sequencing, respectively.

### Analysis of substrates and metabolites

Benzene and toluene were measured by Beckman Gold High-Pressure Liquid Chromatograph (HPLC) (Beckman Coulter, Pasadena, CA) equipped with a Supelco LC-PAH column (Sigma-Aldrich, St. Louis, MO), 25 cm x 4.6 mm filled with 5-µm silica particles. The eluent was acetonitrile in water (60:40 [vol:vol]) (flow rate 0.5 mL/min). Acetate, benzoate, nitrate, and succinate were measured by a Dionex ICS-2000 Ion Chromatograph (IC) (Thermo Scientific, Canton, GA) equipped with a Dionex IonPac AS11-HC anionic resin column (Dionex, Sunnyvale, CA). The eluent was 1 M KOH (flow rate 1.5 mL/min).

### Identification of anaerobic aromatic degradation genes

The genomes of *G. metallireducens* (NC_007517) and *G. daltonii* (NC_011979) were compared using the Nucleotide Basic Local Alignment Search Tool (BLASTN) in the nucleotide collection (nr/nt) database (47). Amino acid sequence alignment was performed using BLASTP (48). Relative locations and similarities of aromatic degradation genes in *G. daltonii* were compared via the KEGG database (49). Organization of genes as operons was determined bioinformatically via DOOR: Database for prOkaryotic OpeRons (50). Operons were analyzed for promoter consensus sequences using *iPro54-PseKNC* (51).

### Total cellular protein profile analysis by SDS-PAGE

*G. daltonii* cells were harvested anaerobically from mid-log growth phase cultures and normalized by OD before centrifugation at room temperature for 20 minutes at 16,000 x g. The cell pellet was resuspended in Laemmli Sample Buffer (Bio-Rad, Hercules, CA) and lysed for 20 minutes in boiling water. The lysate was loaded onto a 20%/4% SDS-PAGE gel and migrated at 40 V through the stacking portion of the gel before running at 90 V through the remainder of the gel. The gels were stained with the Bio-Safe Coomassie G-250 stain (Bio-Rad, Hercules, CA) and de-stained with the Coomassie destain solution.

### Analysis of toluene formation from benzene via cell lysates

To determine if benzene is enzymatically methylated to toluene, whole-cell lysates of *G. daltonii* cultures grown to the mid-log phase on benzene as the carbon source and fumarate or nitrate as the electron acceptor were prepared. Cells were pelleted, resuspended in lysis buffer, and bead-beaten. Aromatic metabolites were extracted from the lysates and were measured by HPLC every 2 minutes for 20 minutes. Fumarate or nitrate was added after 10 minutes. Reactions were halted after 20 minutes by oxygen exposure. A more detailed method is described in Supplemental Material.

### Quantification of metabolite formation in *G. daltonii* whole-cell lysates

To determine if intermediate metabolites are formed during degradation of various aromatic carbon sources, whole-cell lysates of *G. daltonii* cultures grown to the mid-log phase on benzene, toluene, benzoate, and acetate, respectively, and fumarate as the electron acceptor were prepared. Cells were pelleted, washed, and resuspended in deionized water. The pellet was transferred to a glass serum bottle containing glass beads and 1% SDS and bead-beaten for 1 minute. Lysates were filtered using a glass syringe and 0.2-µm PTFE syringe filter. Metabolites were measured by HPLC and IC.

### Complementation assay of σ^54^

Competent *E. coli* strain K12/DH10B and σ^54^- *E. coli* (*rpoN*-deficient) strain JW3169 were transformed with the pCRTOPO2.1 vector (Invitrogen, Carlsbad, CA) ligated with the *bss* operon and *abrB* from *G. daltonii* as per the manufacturer’s instructions. Toluene was added to closed-end capillary tubes stuffed with sterile cotton wool, which was attached to the inside of the Petri dish containing growing *E. coli* cultures. Cells were incubated at 37°C for 7 days and monitored for growth.

### Rapid amplification of 5´ cDNA ends (5´ RACE)

RNA extracted from *G. daltonii* cultures grown on benzene and toluene was treated with 0.1 M DTT and incubated at 42°C for 2 minutes to eliminate the secondary structure. Primers LptC_R and Bss1RaceL (Table S1) were used for first-strand synthesis. cDNA was purified by incubation with 1 M NaOH at 65°C for 20 minutes and GeneJet PCR Purification kit (Thermo Scientific, Pittsburgh, PA). DNA was tailed by incubating with Terminal Transferase (New England Biolabs, Ipswich, MA). PCR was performed using RaceUT and LptC_R or Bss1RaceL primers for 43 cycles with 1 minute of annealing at 54°C and 30 seconds of extension. This step was repeated, replacing the RaceUT primer with RaceU, and for the *rpoN* gene, using the LptC_R_Nest primer (Table S1). PCR products were purified using the GeneJet PCR Purification kit and sequenced via MinION (Oxford Nanopore Technologies, Oxford, UK) with the Rapid Barcoding Kit 96 V14.

### Sequence overlap PCR (SO-PCR) for determination of operonic organization

This method was specifically designed in this study to sequence a total polycistronic mRNA molecule. cDNA was synthesized using *G. daltonii* total RNA and reverse primers RpoN_SO_1_R to RpoN_SO_6_R (Table S1). PCR amplification was performed using the cDNA and each respective reverse and forward primer (RpoN_SO_1_F to RpoN_SO_6_F) (Fig. S1). The amplicons were visualized by agarose gel (Fig. S2) and sequenced via Sanger sequencing. Sequences were aligned and concatenated via Clustal Omega (52) (Fig. S3). A more detailed method is described in the Supplemental Material.

### Purification of AbrB

The *abrB* gene was amplified via the PCR with AbrB_NcoI_F and AbrB_AvrII_R primers (Table S1). The amplicon was double-digested with NcoI and AvrII and cloned into the pETDuet-1 vector. The resulting plasmid was transformed into *E. coli* strain Rosetta 2 (DE3) (Novagen, Madison, WI) as per the manufacturer’s instructions. Transformants were plated on LB media with appropriate antibiotics. 5 mL of LB media with antibiotics was inoculated with a single resulting colony incubated at 37˚C and used to inoculate 500 mL of the antibiotic-containing LB medium. Once OD_600_ reached 0.8, *β*-D-thiogalactopyranoside (IPTG) was added to the culture at 1 mM final concentration. The culture was grown for an additional 4 hours and then centrifuged at 4,500 rpm for 20 minutes. The pellet was resuspended in G-50 buffer (53). Resuspension was sonicated four times with 1-minute pulse on ice. The lysate was centrifuged, and the supernatant was fractionated and purified for AbrB as previously described (53). The concentration was confirmed by Bradford assay (54). Purified AbrB was lyophilized and stored at −80°C.

### DNA-protein binding analysis via electrophoretic mobility shift assay (EMSA)

Lyophilized AbrB protein was dissolved in buffer A as previously described (53). Binding reactions contained promoter DNA, AbrB protein, *raiA* promoter, and the *recA* gene. For σ^70^ binding, reactions contained promoter DNA and σ^70^ saturated RNA polymerase (New England Biolabs, Ipswitch, MA), *bssA* promoter, and the *recA* gene. Reaction was incubated at 25°C for 30 minutes before adding Novex Hi-Density TBE Sample Buffer (Thermo Fisher Scientific, Wilmington, DE). Electrophoresis was performed using a Novex 6% DNA Retardation Gel (Thermo Fisher Scientific, Wilmington, DE). DNA was visualized via ethidium bromide staining and UV illumination. A more detailed method is described in the Supplemental Material.

### *In vitro* expression analysis of AbrB

*G. daltonii* cells were harvested from cultures grown to the mid-log phase on benzene, toluene, or acetate. Growth media was removed, and the cell pellet was washed with anaerobic electroporation buffer (55). The pellet was resuspended in electroporation buffer to 10^11^ cells/mL. DMSO:electroporation buffer (60:40 [vol:vol]) was added to cells. Cells were combined with the plasmid in an electroporation cuvette and electroporated at 25 kV/cm for 100 µs. Transformants were transferred to an anaerobic serum bottle containing freshwater medium. Cells were incubated for 5 hours at 30˚C, and the antibiotic was added. Protein expression was induced via anaerobic IPTG addition. A more detailed method is described in the Supplemental Material.

### Statistical analysis

Unpaired two-tailed Student *t*-tests were performed for statistical analysis at a probability level of *P* < 0.05. For cell density measurement, the results represented the means ± standard errors of triplicate OD_600_ determinations of each sample obtained from triplicate cultures. For analysis of substrates and metabolites, the results represented the means ± standard errors of triplicate HPLC and IC determinations of each cell filtrate sample obtained from triplicate cultures. For gene expression analysis, the results represented the means ± standard errors of the triplicate qRT-PCR determinations of each cDNA sample obtained from triplicate cultures.

## RESULTS AND DISCUSSION

### Anaerobic oxidation of benzene by methylation to toluene

**Fig 1:**
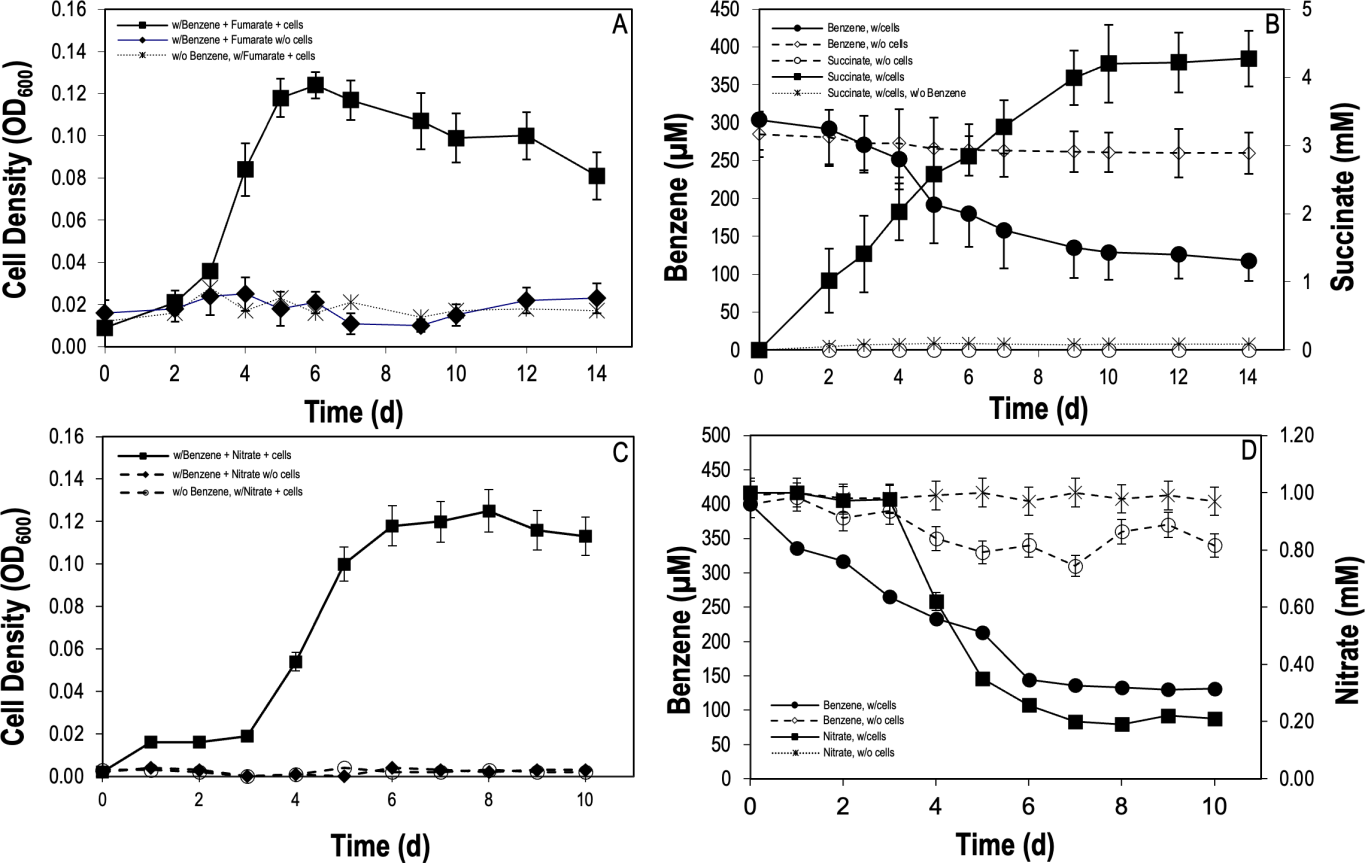
*G. daltonii* utilizes benzene as a sole carbon source. (A) Growth of *G. daltonii* on benzene as a sole carbon source and fumarate as the electron acceptor. (B) Anaerobic benzene oxidation is coupled to reduction of fumarate as the electron acceptor and production of succinate. (C) Growth of *G. daltonii* on benzene as a sole carbon source and nitrate as the electron acceptor. (D) Anaerobic benzene oxidation is coupled to the reduction of nitrate as the electron acceptor. The results represent the means ± standard errors of triplicate OD_600_, HPLC, or IC determinations of each sample obtained from triplicate cultures.

*G. daltonii* was shown to conserve energy for growth on benzene as a sole carbon source/electron donor and fumarate or nitrate as the electron acceptor (Fig. 1). Growth on fumarate was accompanied by the disappearance of benzene and formation of succinate, the product of fumarate reduction (Fig. 1A and B) (56). Electron equivalents recovered in succinate production were shown to be commensurate with the electron equivalents calculated from consumed benzene with a calculated electron recovery of 121.2% (Table S2A). A negative control without benzene was tested, resulting in no growth or succinate accumulation, suggesting that fumarate was not utilized as the substrate (Fig. 1B). Growth on nitrate as an electron acceptor (Fig. 1C and D) showed electron equivalents recovered in nitrate loss to correspond with the electron equivalents of benzene consumed with a calculated electron recovery of 108.6% (Table S2B).

Difficulty in cultivating pure cultures of anaerobic bacteria capable of metabolizing benzene has limited the studies of anaerobic benzene metabolism. Several studies have revealed microorganisms capable of degrading benzene anaerobically; an anaerobic, hypothermophilic, Fe(II)-oxidizing archaeon *F. placidus*, belonging to the family *Archaeoglobaceae* and isolated from hydrothermal vent sediment, carboxylates benzene, converting it to benzoate and completing oxidation via the benzoate degradation pathway (9). *G. metallireducens*, a member of the family *Geobacteraceae*, has been demonstrated to be capable of oxidizing benzene anaerobically by hydroxylation, continuing via the phenol degradation pathway (20). As *G. daltonii* was shown to grow anaerobically on benzene, the metabolic pathway was further investigated in this study.

A well-studied anaerobic toluene degradation operon *bss* and a homolog of *bss*, designated as *hbs* in this study, were identified in *G. daltonii*’s genome (Fig. S4). Genetic alignment of *hbs* and *bss* revealed 76% identity in amino acid sequences.

**Fig 2:**
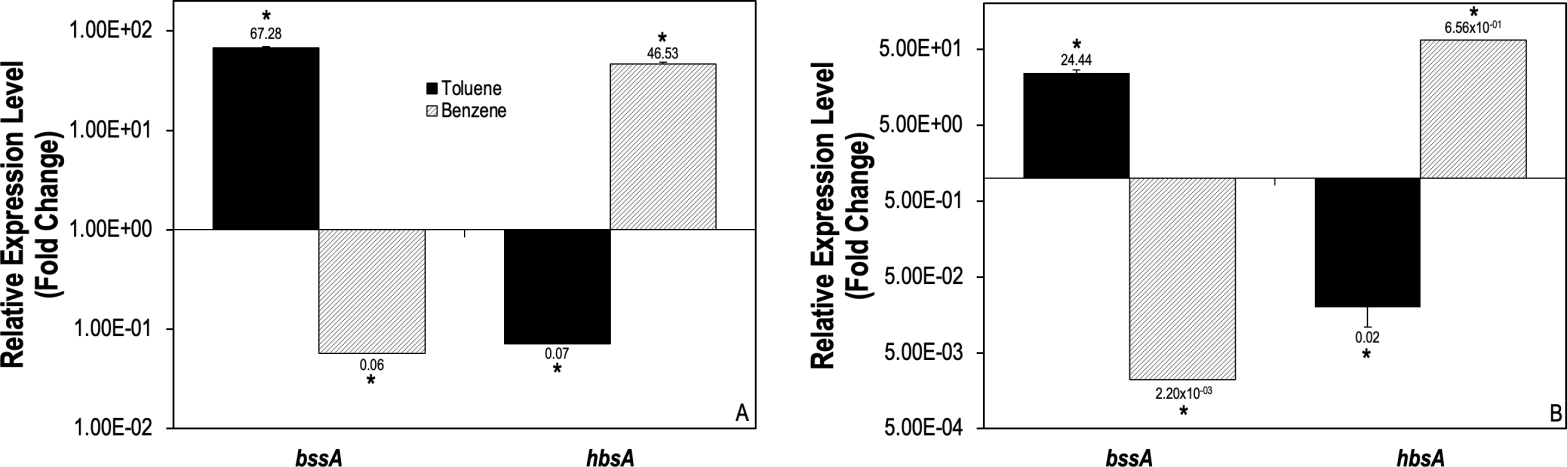
Relative gene expression levels of *bssA* and *hbsA* in *G. daltonii* cultures grown on toluene or benzene as a carbon source and fumarate (**A**) and nitrate (**B**) as the electron acceptors. The fold change shown is relative to the transcript levels during growth on benzoate. Growth on benzoate was used as a negative control. The results represent the means ± standard errors of the triplicate qRT-PCR determinations of each cDNA sample obtained from triplicate cultures (*P < 0.05; as determined by Student’s *t*-test). Significant difference compared to expression during growth on benzoate is indicated by asterisks.

Transcript levels for *bssA* and *hbsA* were quantified (Fig. 2) to elucidate the regulation of genes encoding the active site containing subunits of Bss and Hbs, respectively, under different growth conditions. During growth on toluene and fumarate, *bssA* was upregulated *ca*. twofold compared to growth on benzoate (used as a negative control due to it not being an intermediate in anaerobic toluene metabolism and instead converging with the toluene degradation pathway at the benzoyl-CoA intermediate) (57), while *hbsA* was downregulated *ca*. onefold. However, during growth on benzene, we observed the inverse, i.e., *bssA* was downregulated *ca*. onefold compared to growth on benzoate, whereas *hbsA* was upregulated twofold (Fig. 2A). When grown on benzene and nitrate, *hbsA* was upregulated, but *bssA* was downregulated. During growth on toluene and nitrate, upregulation of *bssA* and downregulation of *hbsA* were observed (Fig. 2B). Downregulation of *bssA* and upregulation of *hbsA* during benzene metabolism, but not during toluene metabolism, indicated that Hbs, and not Bss, was utilized during benzene oxidation.

The *bss* expression during anaerobic toluene degradation has been shown in a number of bacteria, including *Thauera aromatica* (27, 58), *G. metallireducens* (38), *Blastochloris sulfoviridis* (59), sulfate-reducing strain PRTOL1 (60), denitrifying bacterium Strain T (61), and methanogenic and sulfate-reducing cultures (62, 63). A study by Espinoza-Tofalos *et al*. (64) described the expression of *bss*-like genes during BTEX degradation by a bacterial consortium with highly enriched *Geobacter* species.

**Fig 3:**
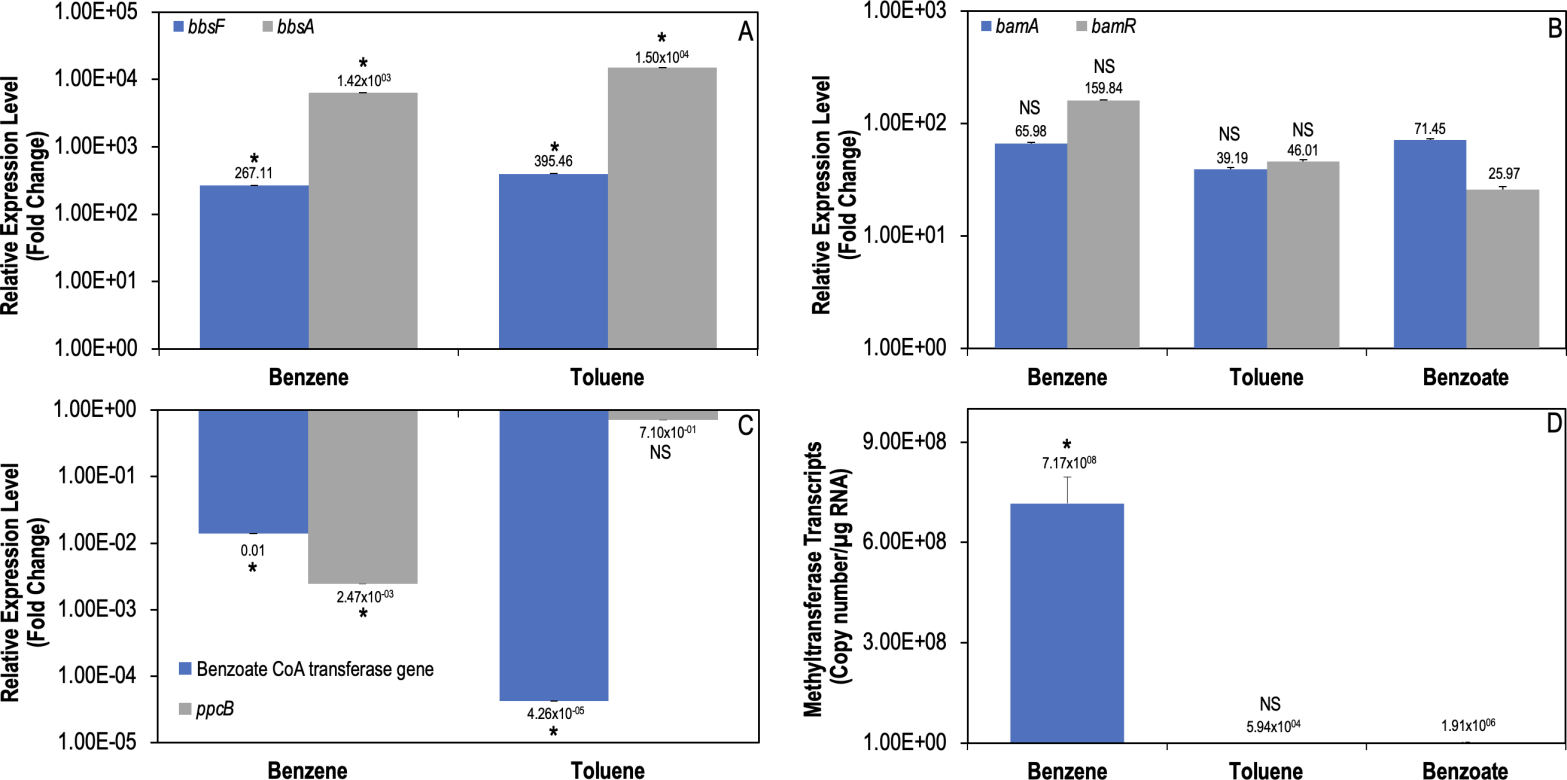
Relative expression of genes involved in anaerobic aromatic oxidation in *G. daltonii*. (A) Relative expression levels of *bbsF* and *bbsA* during benzene and toluene degradation. The fold change is relative to the transcript level during growth on benzoate as a negative control. (B) Relative expression levels of *bamA* and *bamR* during benzene, toluene, and benzoate degradation. The fold change shown is relative to the transcript level during growth on acetate as a negative control. (C) Relative expression levels of the benzoate CoA transferase gene and *ppcB* during benzene and toluene degradation. (D) Transcript levels of the methyltransferase gene during benzene, toluene, and benzoate degradation. The fold change is relative to the transcript level during growth on benzoate as a negative control. The results represent the means ± standard errors of the triplicate qRT-PCR determinations of each cDNA sample obtained from triplicate cultures (*P < 0.05; NS = not significant; as determined by Student’s *t*-test). Significant differences compared to expression during growth on benzoate is indicated by the asterisks.

To determine whether the benzene and toluene degradation pathways converge into a single metabolic pathway, the total cellular protein profile was visualized via SDS-PAGE (Fig. S5). *G. daltonii* cultures grown on toluene or benzene expressed proteins that correspond to the size of two succinyl-CoA:benzylsuccinate CoA-transferase subunits (BbsE and BbsF) and (R)-benzylsuccinyl-CoA dehydrogenase (BbsG) (Table S3), all of which are encoded by the *bbsEFG* operon and are utilized for β-oxidation of benzylsuccinate during anaerobic toluene degradation (65). These proteins were not expressed in cultures grown on benzoate, indicating that an alternative pathway was utilized during benzene metabolism compared to benzoate metabolism, and therefore benzene was not carboxylated to benzoate. Expression of succinyl-CoA:benzylsuccinate CoA-transferase was confirmed via transcript level analysis for the *bbsF* gene (Fig. 3A). The upregulation of *bbsF* during growth on benzene and toluene indicated that the same metabolic pathway was utilized during degradation of both compounds.

Further analysis was conducted to determine if benzene degradation follows a canonical toluene degradation pathway after methylation to toluene. The *bbsA* gene, utilized prior to toluene degradation pathway convergence with the anaerobic benzoate degradation pathway, was upregulated during benzene oxidation (Fig. 3A). Transcript levels for *bamR*, encoding the enzyme responsible for dearomatizing the benzene ring in toluene degradation, and *bamA,* the gene encoding the enzyme responsible for opening the dearomatized benzene ring in the same pathways, showed no significant differences in regulation, regardless of cultures grown on toluene, benzene, or benzoate (Fig. 3B) (66, 67), indicating that convergence of the benzene, toluene, and benzoate pathways occurred and that degradation of all three compounds continued down a single common pathway. As these genes are constituents of the anaerobic toluene degradation pathway, it suggests that benzene was methylated to toluene and continued oxidation via the toluene degradation pathway. To further confirm that the toluene degradation pathway and no other anaerobic benzene degradation pathways were utilized during benzene degradation in *G. daltonii*, genes constituent to other anaerobic benzene-degrading pathways were examined. The *ppcB* gene, encoding for phenylphosphate carboxylase, an enzyme integral to hydroxylation of benzene during anaerobic benzene oxidation in *G. metallireducens* (68), was downregulated, indicating that benzene degradation was not occurring via the anaerobic phenol degradation pathway (Fig. 3C). Geob_2194, the gene encoding for benzoate-CoA transferase, an enzyme essential to carboxylation of benzene during anaerobic benzene degradation in *F. placidus* (9), was also downregulated, further indicating benzene degradation was not occurring via the benzoate degradation pathway (Fig. 3C). The utilization of the toluene degradation pathway and not the carboxylating or hydroxylating pathways indicated that methylation of benzene to toluene was occurring during anaerobic benzene oxidation in *G. daltonii*. The presence of putative genes known to be involved in alternative benzene-degrading pathways (Table S4) indicates the possibility of alternative benzene activation pathways utilized by *G. daltonii* under different growth conditions.

**Fig 4:**
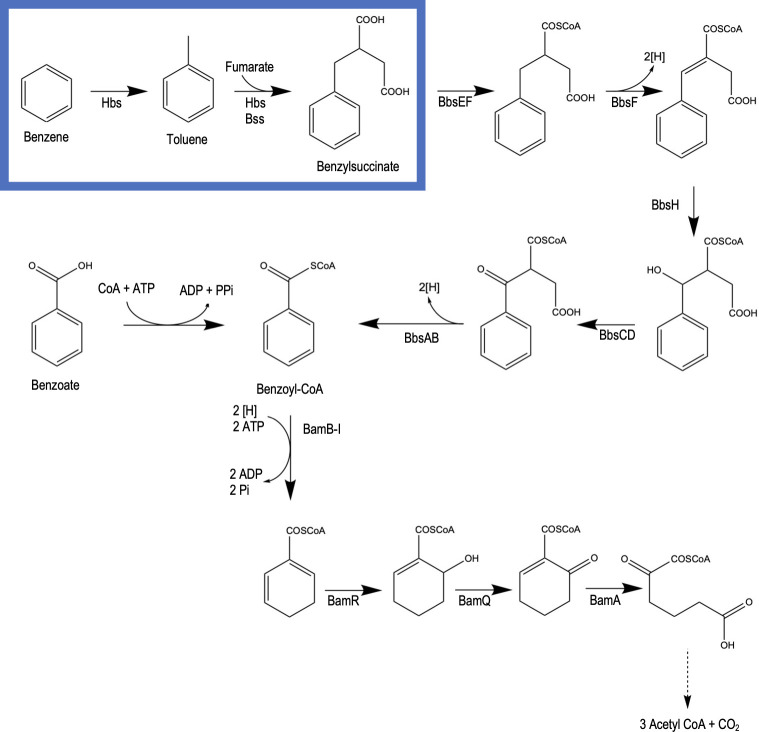
Proposed anaerobic benzene degradation pathway in *G. daltonii*. Benzene is methylated to toluene, to which fumarate is added via Hbs (during benzene degradation) or Bss (during toluene degradation) before continuing down the toluene degradation pathway (indicated by box). Both benzene and toluene pathways converge with the traditional anaerobic benzoate degradation pathway, all of which generate the metabolite benzoyl-CoA.

Functional site analysis of Hbs via ExPASy’s ScanProsite (69) identified that an S-adenosylmethionine (SAM) site is present on the activating subunit Hbs activase (encoded by *hbsD*) (Fig. S6A). SAM sites have two functions. They can form a radical, which is generated in Bss and abstracted into the BssA active site (28). SAM sites also can perform methylation, offering a potential mechanism that *G. daltonii* could employ to perform the first step of activation of benzene to toluene (70, 71). A methyltransferase gene would be necessary for methylation to occur via SAM site as it would facilitate transfer of the methyl group to the benzene substrate (71). Analysis of the *G. daltonii* genome revealed the putative methyltransferase gene (Geob_0241). Upregulation of Geob_0241 during benzene oxidation, but not during toluene or benzoate oxidation (Fig. 3D), suggested a pathway by which a resident SAM site could methylate benzene, providing a method of methylation as a benzene activation mechanism. We proposed that anaerobic benzene oxidation in *G. daltonii* begins by methylation, followed by fumarate addition to the newly formed toluene by Hbs, after which oxidation continues down the toluene degradation pathway, as outlined in Fig. 4. To elucidate this, we investigated whether anaerobic benzene oxidation in *G. daltonii* results in toluene formation.

**Fig 5:**
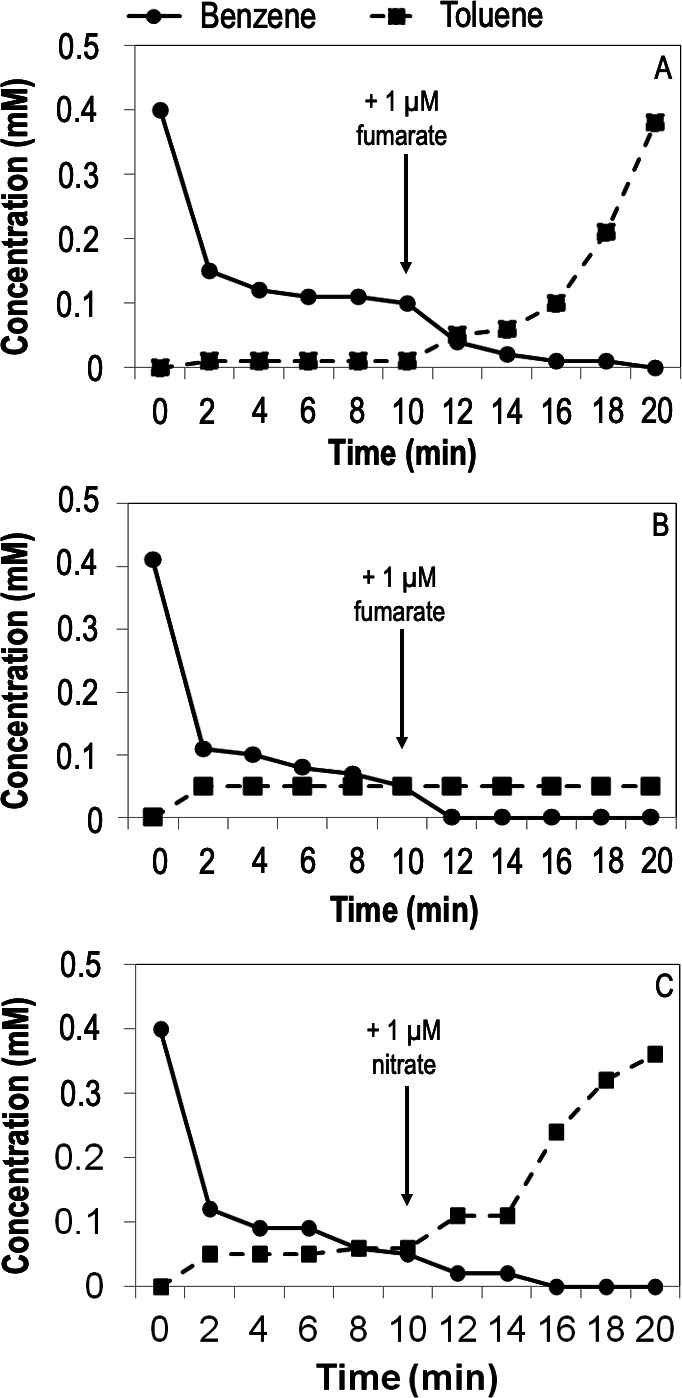
Degradation of benzene in *G. daltonii* whole-cell lysates correlates with toluene formation. (A) Lysates were from *G. daltonii* cultures grown on fumarate as an electron acceptor. (B) Lysates were from *G. daltonii* cultures grown on nitrate as an electron acceptor. (C) Lysates were from *G. daltonii* cultures grown on nitrate as an electron acceptor. Arrows indicate addition of fumarate or nitrate after 10 minutes.

*G. daltonii* whole-cell lysates from benzene-oxidizing, fumarate-reducing cultures showed benzene loss (Fig. 5A). Fumarate addition resulted in further loss of benzene and accumulation of toluene equal to 95% of the original benzene concentration. To determine whether fumarate was utilized as an electron acceptor or as a co-substrate of Bss, this experiment was performed with the whole-cell lysates obtained from benzene-degrading *G. daltonii* cultures grown on nitrate as the electron acceptor. Toluene accumulation halted after *ca*. 5 minutes, with fumarate addition failing to increase the activity (Fig. 5B). However, nitrate addition caused continued benzene degradation and toluene accumulation equal to *ca*. 90% of the original benzene concentration (Fig. 5C). The addition of nitrate rather than fumarate suggested that it was necessary for additional electron acceptor to be present to ensure that the reactions catalyzing the methylation of benzene to toluene could proceed forward without enzymes becoming saturated. Toluene accumulation appeared to be delayed until after benzene degradation had already been established. No other aromatic intermediate was detected, suggesting that early toluene accumulation is sequestered within the lysate; as methylation is a multi-enzyme process (utilizing an SAM site as well as a methyltransferase) (71), it is likely that the molecule is “trapped,” either within the active sites of these enzymes or within another coenzyme pertinent to methylation of benzene. The toluene accumulation and halting of further degradation can be explained by a likely multi-pronged process performed by Hbs activase; the subunit is first utilized for methylation to toluene using its constituent SAM site (71), after which the same SAM site would be utilized to generate a radical for fumarate addition to the toluene for benzylsuccinate formation, as is performed by Bss during toluene degradation (72). A limited concentration of Hbs activase used for two different enzymatic mechanisms (methylation followed by radical generation) would cause a bottleneck, resulting in toluene accumulation (73–75).

**Fig 6:**
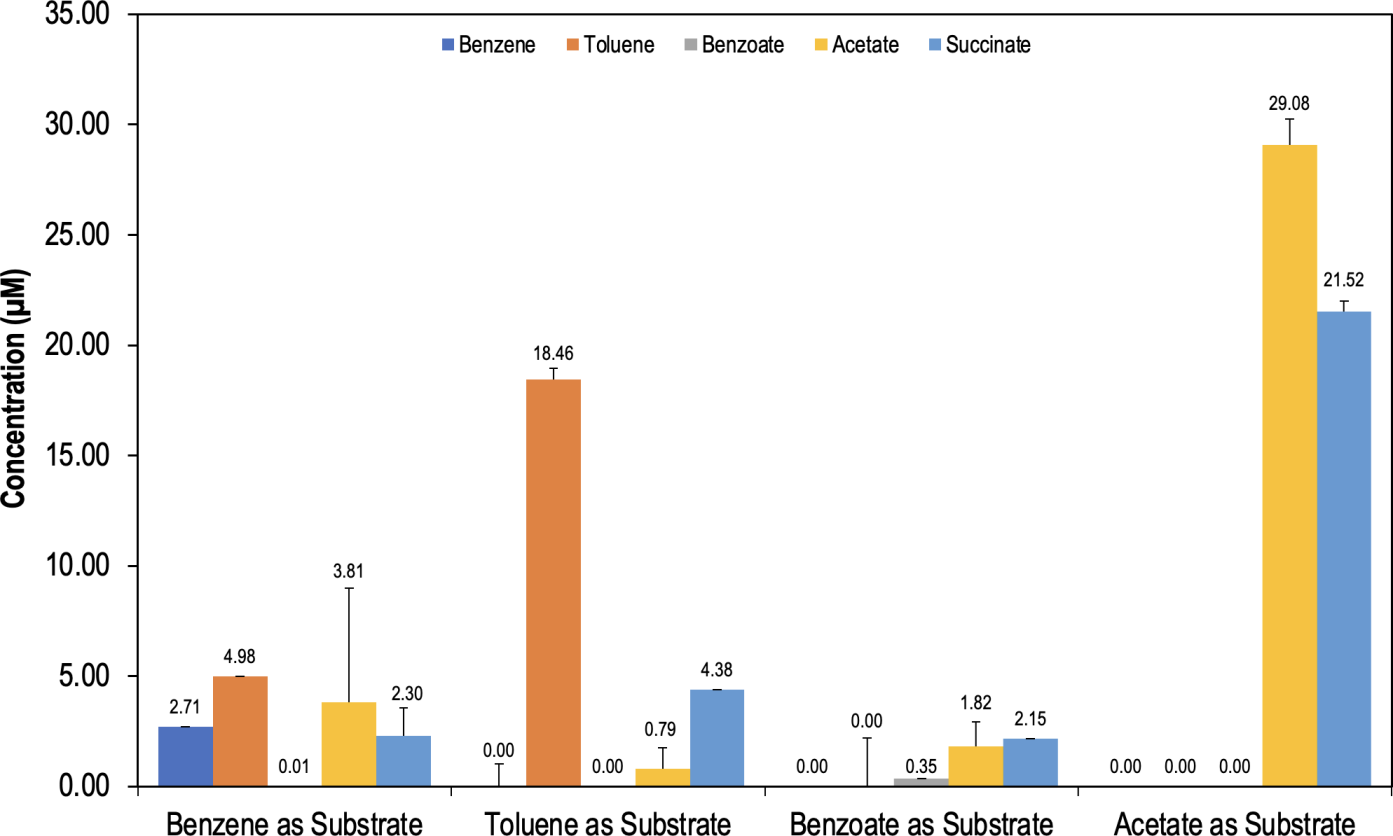
Accumulation of intermediate metabolites in *G. daltonii* whole-cell lysates from cultures at the mid-log growth phase during oxidation of various carbon sources. Succinate is a product of fumarate reduction. The results represent the means ± standard errors of triplicate HPLC and IC determinations of each sample obtained from triplicate cultures.

To determine whether benzene degradation is coupled to toluene accumulation *in vivo*, various metabolites were identified and quantified in the whole-cell lysates from *G. daltonii* cultures grown on acetate, benzene, toluene, and benzoate as sole carbon sources, respectively, and fumarate as the electron acceptor (Fig. 6). The acetate-degrading *G. daltonii* cultures, used as a non-aromatic control, showed accumulation of acetate and succinate. No benzene, toluene, or benzoate were detected in acetate-degrading cultures. Accumulation of benzene, toluene, succinate, and acetate was detected in benzene-degrading cultures. Accumulation of toluene, succinate, and acetate was detected in toluene-degrading cultures. Toluene accumulation in benzene-degrading cultures supports the hypothesis that benzene is methylated to toluene. Benzoate was detected in the benzoate-degrading cultures, along with succinate and acetate. Benzoate accumulation in benzoate-degrading cultures, but not in toluene- or benzene-degrading cultures, is expected as the toluene and benzene degradation pathways in *G. daltonii* are not suggested to form benzoate as an intermediate metabolite, but instead to converge with the benzoate degradation pathway, as proposed in Fig. 4 (58).

The detection of toluene, succinate, and acetate in both benzene- and toluene-degrading *G. daltonii* cultures indicated that the same metabolic pathways were utilized during benzene or toluene degradation. Detection of toluene in benzene-degrading *G. daltonii* cultures indicated that, *in vivo*, toluene was formed.

Anaerobic benzene oxidation via methylation has been proposed as an alternative activation mechanism to carboxylation and hydroxylation (76, 77). By converging benzene degradation with toluene degradation, *G. daltonii* utilizes a conservative metabolic pathway, creating a single route for degradation of both recalcitrant compounds. This not only permits simultaneous degradation of these chemicals, but requires no extra resources be expended by *G. daltonii* during degradation of both compounds, a signature of metabolic specialization (78).

Due to its thermodynamic stability, benzene degradation in the absence of oxygen necessitates the use of a radical for methylation (76). SAM sites are common co-substrates capable of activating inert C-H compounds and methylating aromatic rings (71). Our findings suggested that the activating subunit of benzylsuccinate synthase, Bss activase, and the activating subunit of 3-hydroxybenzylsuccinate synthase, Hbs activase, contain SAM sites (Fig. S6). SAM sites have the ability to form radicals (as is the case with pyruvate formate lyase activating enzymes like Bss) (79) and methylate aromatic rings (71). The presence of these SAM sites and the expression of methyltransferase gene (Fig. 3D) suggested an enzymatic mechanism by which *G. daltonii* could methylate benzene to toluene.

### Regulation of anaerobic aromatic oxidation by *G. daltonii*

Directly upstream and in opposite orientation of *G. daltonii*’s *bss* and *hbs* operons are genes encoding for σ^54^-dependent Fis family transcriptional regulators 77% identical in amino acid sequence to *G. metallireducens* σ^54^ enhancer binding proteins*,* indicating *G. daltonii* utilizes a σ^54^-dependent regulation system for the *bss* and *hbs* operons (Fig. S7) (80). Using the program *iPro54-PseKNC* (51), regions upstream of the *hbs* and *bss* operons were screened for potential sigma factor binding sites, revealing σ^54^ consensus sequences. Analysis of those regions indicated the presence of the mandatory GG and GC elements at −12 and −24 (81), respectively, as well as other less highly conserved elements. These predicted sequences were confirmed *in vitro* using 5´ RACE (Fig. S7), which revealed the consensus sequence TGGCATGTCTTCTGCTAAATAGTCC upstream of the *bss* operon and TGGCATGGCTCCTGCTATCAATATC upstream of the *hbs* operon, demonstrating the inclusion of the GG and GC elements within each promoter.

*E. coli* strain K12/DH10B transformed to include the *bss* operon and grown in the presence of toluene showed colony formation after 7 days, while untransformed cells failed to grow (Fig. S8). *E. coli* strain JW3169 lacking a functional σ^54^ did not grow in the presence of toluene, even after the *bss* operon was transformed into *E. coli*. However, complementation of the *rpoN* gene (encoding for σ^54^) from *G. daltonii* combined with the *bss* operon restored the ability to grow in the presence of toluene, indicating that the *bss* operon is regulated by σ^54^. The ability of the transformed *E. coli* to grow and form colonies suggested that the *bss* genes were actively transcribed and that their protein products were able to catalyze the conversion of toluene (toxic to *E. coli*) to benzylsuccinate (not toxic to *E. coli*). The inability of σ^54^- *E. coli* to grow using *bss* unless *rpoN* was complemented back into it indicates that, to be active, the *bss* genes require σ^54^.

The σ^54^ is an alternate transcription factor to σ^70^, responsible for the control of a subset of genes (82). Initially, σ^54^ was considered to be associated with nitrogen levels (and therefore was originally referred to as σ^N^) (83). However, the utilization of σ^54^ in the metabolism of alternative carbon sources including aromatic degradation has been reported in *G. metallireducens* and other bacteria (84, 85). A unique aspect of σ^54^ is its mechanism after recruitment of RNA polymerase (RNAP); unlike σ^70^, which can initiate transcription after binding to RNAP, RNAP-σ^54^ remains inactive until an activator protein binds to enhancer-like sequences (86). By providing this kind of regulation for those genes under σ^54^ promotion, *G. daltonii* can conserve resources by strictly controlling gene expression during changing environmental conditions. While the ability to anaerobically oxidize benzene and toluene is beneficial to *G. daltonii*, allowing it to thrive in contaminated environments, this comes at a high energetic cost that may not be ideal for every condition. We hypothesized that the σ^54^ utilization in regulation of anaerobic aromatic oxidation allows *G. daltonii* to quickly prevent the expression of those aromatic degradation genes, preserving vital resources that could be used for degradation of other, more easily degradable substrates (87).

*In silico* analysis via *DOOR: database of prokaryotic operons* suggested that *rpoN* is arranged as an operon along with the *lptCAB* genes, which were predicted to encode for part of *G. daltonii*’s lipopolysaccharide transport system (50). This was confirmed *in vitro* via SO-PCR. The arrangement of *rpoN* as an operon along with *lptCAB* (Fig. S3) indicated that promotion of the entire operon is controlled by a promoter region upstream of the *lptC* gene. Thus, modulation of the *rpoN* gene would occur upstream of the *lptC* gene.

**Fig 7:**
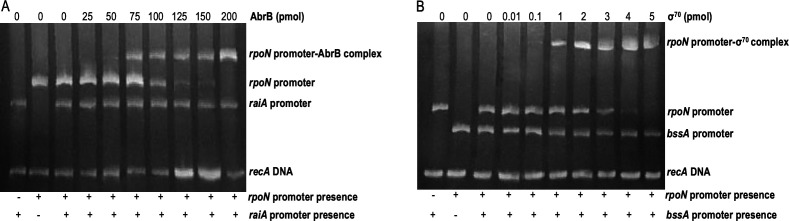
Interactions between *rpoN* promoter and expression factors. Amplicons of the *rpoN* promoter region and purified σ^70^ (**A**) or AbrB (**B**) were analyzed by EMSA. The *bssA* promoter (**A**) or *raiA* promoter (**B**) was included in each reaction as a competitor promoter, except in those lanes containing no expression factor. A 150-bp fragment of the *recA* gene was included in each reaction to eliminate any nonspecific binding reactions.

*In silico* analysis of the *rpoN* operon via Virtual Footprint Bacterial Regulon Analyzer and Bacterial Promoter Prediction (88, 89) indicated the presence of AbrB and σ^70^ binding sites in the *rpoN* operon promoter region. Binding of σ^70^ was confirmed via EMSA (Fig. 7A). As the concentration of σ^70^ was increased, the band representing the *rpoN* operon promoter shifted to a higher molecular weight, while no shift in the weight of the *bss* promoter or the *recA* segment was observed, indicating that σ^70^ binds selectively to the *rpoN* operon promoter region, with no observed binding occurring between the *bss* operon promoter and σ^70^, regardless of the σ^70^ concentration.

To confirm the presence of the AbrB binding site, the inclusion of an AbrB pseudo-consensus sequence upstream of the *rpoN* operon was confirmed by 5´ RACE, revealing the +1 transcription start site to be 184 bp upstream of the *lptC* ATG start codon. The motif TGGCA-TGGAGA-TTGAA appeared 40 bp upstream of the +1-start site, closely matching the AbrB pseudo-consensus binding sequence TGGNA-XXXXX-TGGNA (Fig. S9) (32). AbrB consensus binding regions do not have to precisely match the pseudo-consensus sequence or lie directly between the −37 to −95 nucleotides upstream of the +1 transcription start site (90). To determine the physiological evidence of AbrB involvement in the regulation of *rpoN,* binding of AbrB to the promoter region of the *rpoN* operon was tested by EMSA (Fig. 7B). As the concentration of AbrB was increased, the band representative of the *rpoN* operon shifted to a higher molecular weight, while no shift in the weight of the *raiA* or *recA* segments was observed, indicating that AbrB binds selectively to the *rpoN* operon promoter region.

**Fig 8:**
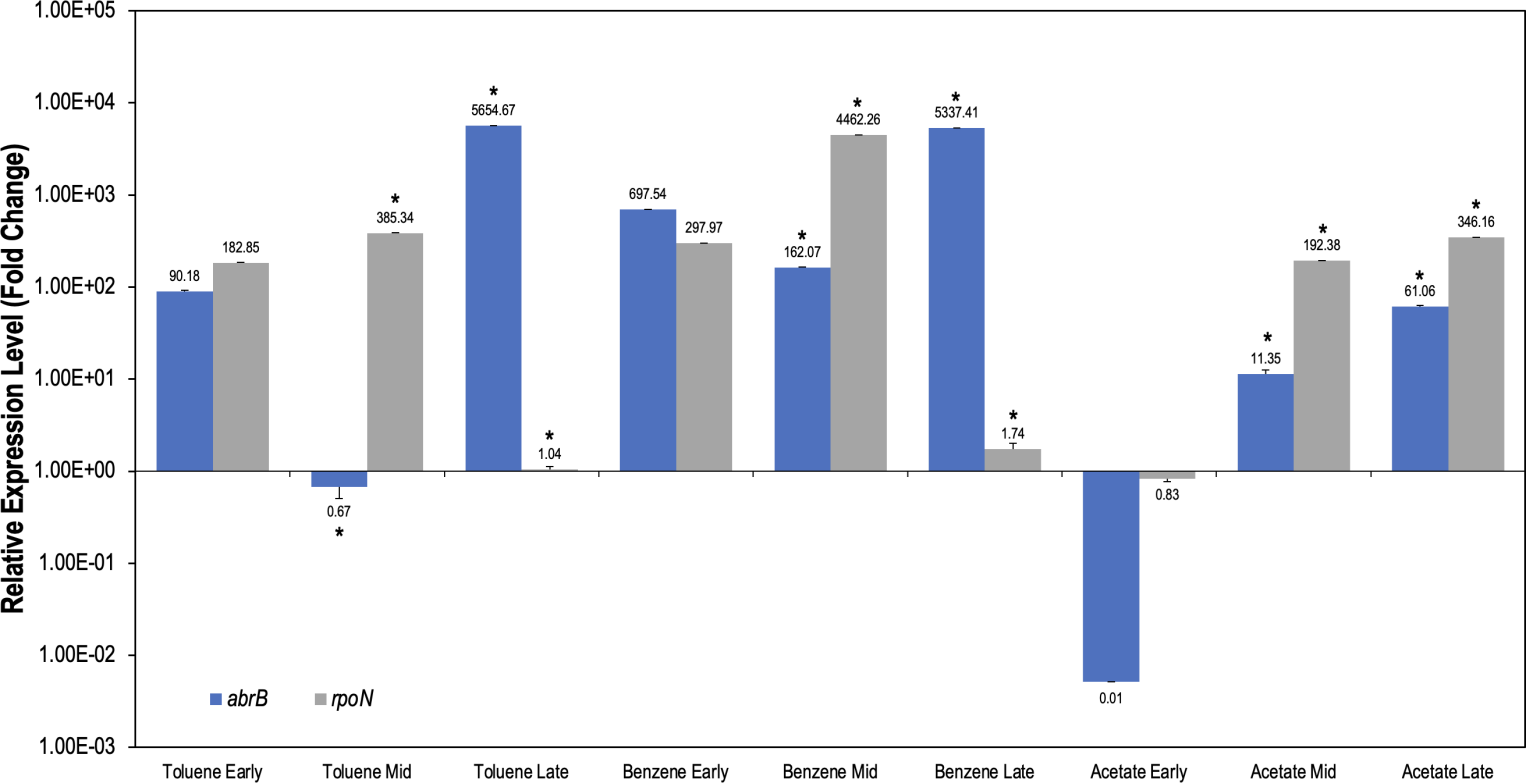
Relative gene expression levels of *abrB* and *rpoN* in *G. daltonii* cultures grown on toluene, benzene, or acetate as the sole carbon source. Expression was analyzed using total RNA from cultures grown at early-, mid-, and late-log phases. Cultures grown on acetate were used as a negative control. The fold change is relative to the transcript levels of a housekeeping gene *recA*. Upregulation of *abrB* was compared to regulation of *rpoN* for modulation. The results represent the means ± standard errors of the triplicate qRT-PCR determinations of each cDNA sample obtained from triplicate cultures (*P < 0.05; NS = not significant as determined by Student’s *t*-test). Significant difference compared to expression during the early-log phase is indicated by asterisks.

The mechanism utilized by AbrB is not fully understood, but it has been well reported that its interaction causes targeted gene repression (35, 91). To determine interactions between AbrB and *rpoN*, gene expression was analyzed in *G. daltonii* cultures grown on toluene, benzene, and acetate, respectively, during early-log, mid-log, and late-log growth phases (Fig. 8). As benzene- and toluene-degrading cultures transitioned from early-log to mid-log growth phases, *abrB* expression was reduced twofold (on toluene) or *ca*. onefold (on benzene) and *rpoN* expression increased 0.5-fold (on toluene) or onefold (on benzene). During the late-log phase, *abrB* was upregulated *ca*. twofold (on toluene) or onefold (on benzene) and *rpoN* was reduced twofold (on benzene and toluene) compared to the early-log phase. In acetate-degrading cultures transitioning from early- to mid-log phases, *rpoN* and *abrB* were upregulated threefold and *ca*. twofold, respectively. As acetate-degrading cultures transitioned to the late-log phase, *abrB* and *rpoN* were upregulated fourfold and twofold, respectively, compared to the early-log phase.

Upregulation of *rpoN* at the mid-log growth phase during aromatic degradation was expected, as we demonstrated by the complementation assay described above; σ^54^ was necessary for the expression of *bss* and *hbs*. Without *rpoN* expression, there would be no system for expression of those operons, preventing aromatic degradation. The upregulation of *abrB* during the late-log growth phase was also expected, as AbrB is a transition-state regulator, and late-log growth corresponds to the transition from the logarithmic phase into the stationary phase. The upregulation of both *abrB* and *rpoN* during the early-log phase indicated a secondary system for *abrB* activation outside of a direct gene repression under its control. For example, the BamVW two-component signaling system has been reported to modulate pathways in the presence of aromatic compounds in *G. metallireducens* and provides a potential secondary mechanism for the expression of genes involved in the regulation of aromatic degradation (31). Similar mechanisms were shown in AbrB-utilizing *B. subtillis* (92). The arrangement of *lptCAB* on the operon along with *rpoN* represented the pleiotropic nature of AbrB and indicated other mechanisms under AbrB’s control necessary for *G. daltonii* to thrive, such as iron uptake and biofilm formation (93). The biofilm is a matrix composed of extracellular polymeric substances created by microbes to aid in synergistic relationships vital to survival (94). Increase in biofilm formation under unfavorable conditions allows for *G. daltonii* to grow to higher concentrations and allow for “flocking” to occur (95). Future studies of *G. daltonii* should investigate the effect of *abrB* modulation on biofilm formation.

The upregulation of *abrB* and *rpoN* during degradation of acetate, a non-aromatic compound, further indicated that the mechanism by which *abrB* and *rpoN* are modulated may be under the control of a system that is active only when aromatics are detected by *G. daltonii* (such as by the BamVW two-component signaling system) (31). By allowing both *rpoN* and *abrB* to be moderately regulated at various phases of logarithmic growth during acetate degradation, *G. daltonii* could potentially keep itself in a state of “readiness” so that genes can be quickly modulated when external conditions change, similar to a mechanism involving AbrB and the two-component signaling system PhoPR in *B. amyloliquefaciens* (35). A similar “state of readiness” system was shown in *Pseudomonas aeruginosa* during the stringent stress response, which stimulated the global transcript regulators and downstream effectors, allowing for rapid adaptation to stressful conditions (96). In our study, *G. daltonii* cultures were grown strictly on a single substrate. However, in nature, the availability of various substrates is dynamic, and therefore the ability to modulate which substrate is degraded becomes more essential as the cost of degradation changes (97).

### Role of AbrB in anaerobic aromatic degradation by *G. daltonii*

The findings in this study suggested that the initial steps in anaerobic benzene and toluene oxidation were performed by Hbs and Bss, respectively, and that the encoding operons were promoted by the transcription factor σ^54^. Therefore, by preventing the expression of σ^54^, benzene and toluene degradation would cease as the genes encoding for Bss and Hbs would no longer be promotable, disabling the benzene and toluene metabolic pathways. To confirm this, *G. daltonii* cells were transformed with an IPTG-inducible *abrB* gene.

**Fig 9:**
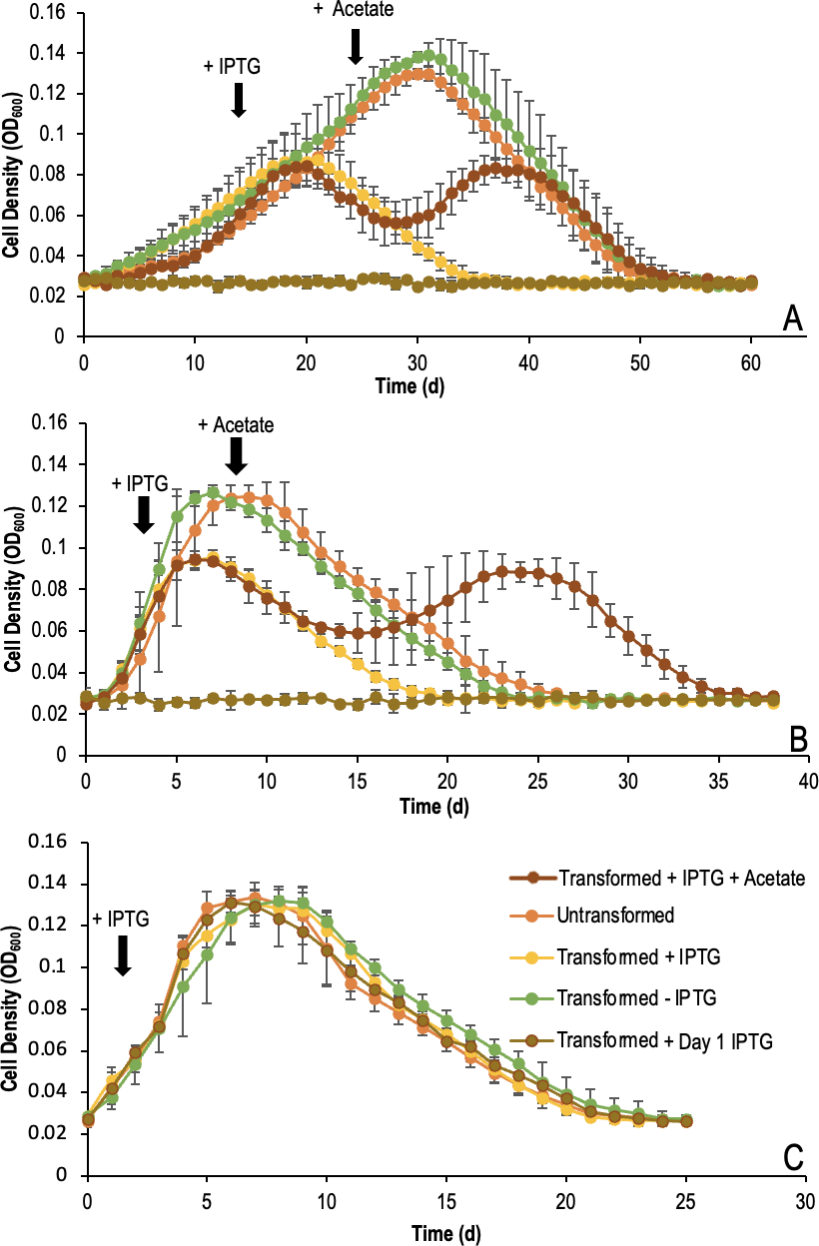
Growth characteristics of *G. daltonii* cells transformed with IPTG-inducible *abrB*. (A) Cells grown on benzene as the sole carbon source. Arrow indicates the addition of IPTG and acetate after 14 and 24 days of cultivation, respectively. (B) Cells grown on toluene as sole carbon source. The arrow indicates addition of IPTG and acetate after 3 and 8 days of cultivation, respectively. (C) Cells grown on acetate as the sole carbon source. The arrow indicates IPTG addition after 2 days. The results represented the means ± standard errors of triplicate OD_600_ determinations of each sample obtained from triplicate cultures.

The growth of transformed *G. daltonii* cells on benzene (Fig. 9A) and toluene (Fig. 9B) reached logarithmic growth at an equal rate to untransformed cells, except when IPTG was added directly after transformation, yielding cells that failed to grow. Transformed cultures with no IPTG addition exhibited growth comparable to that of untransformed cells. Once transformed cultures were spiked with IPTG, they showed truncated logarithmic growth, transitioning to the stationary phase over a period of around 1 (benzene) or 2 (toluene) days. Three (toluene) or 5 (benzene) days after IPTG addition, cultures entered the death phase, unless an alternative, non-aromatic electron source (acetate) was added, causing cultures to transition to a second phase of logarithmic growth *ca*. 4 days later. The second phase of logarithmic growth reached a maximum OD of 0.084 (benzene) or 0.089 (toluene), lasting approximately 7 days before returning to the stationary phase, followed by the death phase until cultures were no longer active. *G. daltonii* cells from the acetate-degrading culture (Fig. 9C) grew comparably to untransformed cells, reaching approximately the same OD and transitioning through all growth phases, regardless of IPTG addition.

**Fig 10:**
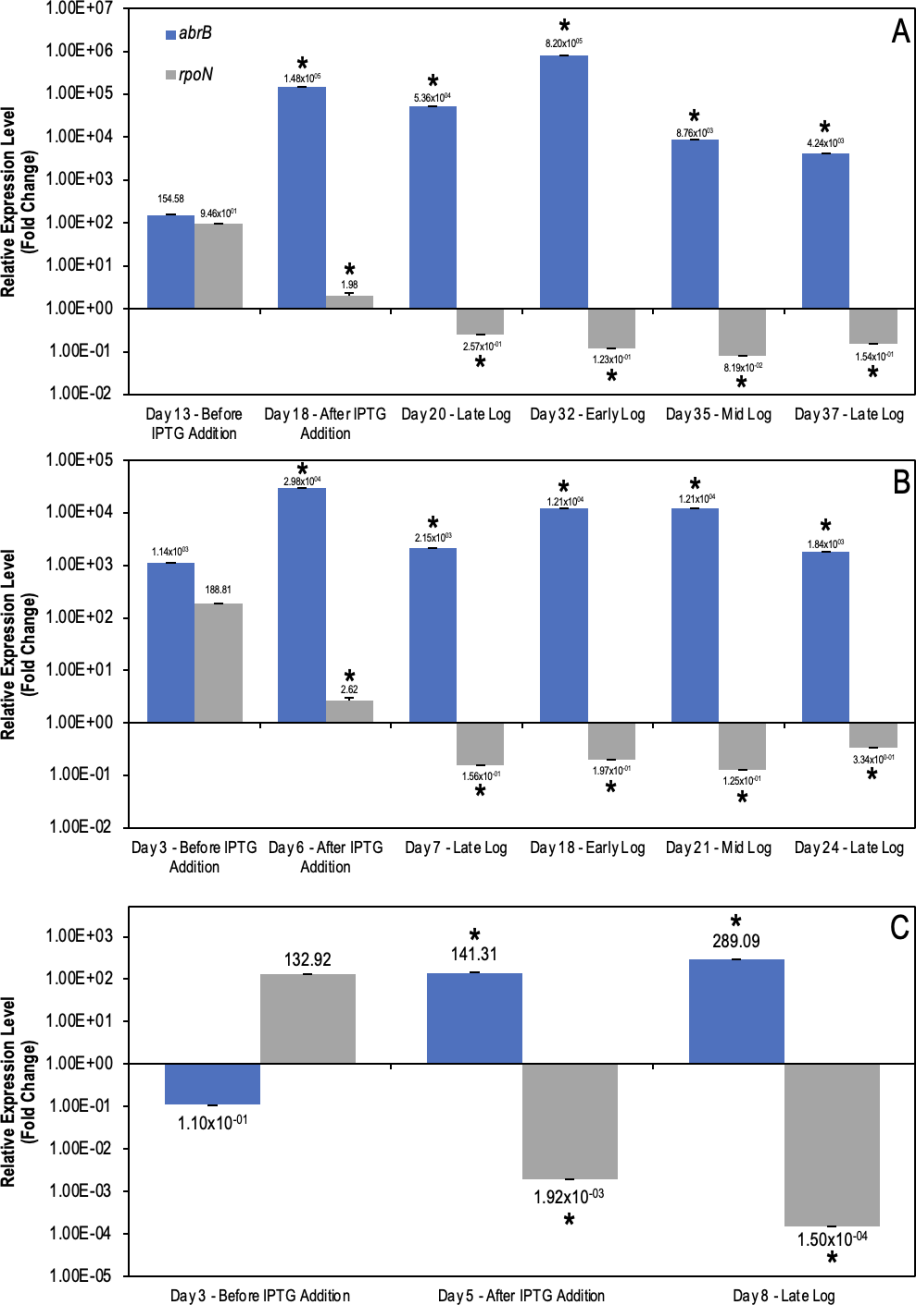
Relative gene expression of *abrB* and *rpoN* in *G. daltonii* cultures transformed with plasmids containing IPTG-inducible *abrB*. Cultures were grown on benzene (**A**), toluene (**B**), or acetate (**C**) as the sole carbon source. The fold change shown is relative to the transcript level of a housekeeping gene *recA*. The results represented the means ± standard errors of the triplicate qRT-PCR determinations of each cDNA sample obtained from triplicate cultures (*P < 0.05; as determined by Student’s *t*-test). Significant differences compared to expression before IPTG addition are indicated by asterisks.

Relative expression of *abrB* and *rpoN* during the lag phase, mid-log phase, and stationary phase was determined in transformed *G. daltonii*. Cultures grown on benzene and toluene before IPTG addition exhibited similar expression profiles of *abrB* and *rpoN* as untransformed cells (Fig. 10A and B). However, after IPTG addition, *abrB* expression increased, to levels threefold higher under benzene-degrading conditions, and 1.5-fold higher under toluene-degrading conditions. Expression of *rpoN* was downregulated *ca*. 1.5-fold under benzene- and toluene-degrading conditions. The *abrB* and *rpoN* expression remained at these levels throughout the lifespan of each culture. Expression profiles of transformants grown on acetate (Fig. 10C) showed threefold higher expression of *abrB*, while *rpoN* expression decreased fourfold.

Our finding that *abrB* expression was induced by IPTG addition suggests that intracellular concentration of AbrB was increased throughout each culture’s growth cycle, a phenomenon that was reported to occur in *B. licheniformis* as *abrB* expression was modulated (98). When intracellular AbrB concentration increased, *rpoN* expression was repressed, causing σ^54^ to be unavailable for *bss* and *hbs* expression. The transition to a second-log growth phase after acetate addition by aromatic-degrading cultures indicated that *abrB* expression only affected the aromatic degradation metabolic pathways. By suppressing aromatic degradation, *G. daltonii* can prevent aromatic oxidation and can quickly transition to degradation of other less recalcitrant substrates. AbrB is known to modulate a number of different survival-associated processes in *B. subtilis* and *B. anthracis* (99). In nature, it is unlikely that aromatic hydrocarbons are the sole carbon source available; this mechanism would allow *G. daltonii* to quickly switch strategies as carbon source availability changes. By switching from aromatic hydrocarbon to non-aromatic compound degradation, *G. daltonii* can direct resources to better thrive in unfavorable environmental conditions. Burton *et al*. (33) concluded that upregulation of sigma factors can be toxic, and by modulating those sigma factors, AbrB counteracts that toxicity. A similar mechanism in *G. daltonii* suggested that, *in vivo*, uninhibited expression of *rpoN* is detrimental to cell growth by preventing it from repressing energetically unfavorable processes.

A study by Tolibia *et al*. (100) examined indirect modulation of *abrB* in *B. subtilis* as a potential tool for enhanced production yield of commercial interest metabolites by genetic engineering. Our study specifically focused on the effects of AbrB on anaerobic benzene and toluene degradation pathways. However, the pleiotropic nature of *abrB* suggested that there are other potential avenues for enhancing *G. daltonii* growth, including by modulating biofilm formation (101). The ability to bioengineer *G. daltonii* to modulate these pathways could be leveraged for improved bioremediation of BTEX contaminated groundwater, expediting complete remediation.

### Conclusions

This study investigated anaerobic microbial degradation pathways of the persistent groundwater contaminant benzene to predict natural attenuation and provide the basis for improved bioremediation strategies more accurately. Genomic analysis of *G. daltonii*, isolated from contaminated groundwater, revealed the presence of putative aromatic-degrading genes, and *G. daltonii* was subsequently shown to conserve energy for growth on benzene as the sole electron donor and fumarate or nitrate as the electron acceptor. Our findings suggested that benzene is first activated by methylation to toluene in *G. daltonii*, a unique mechanism that has not been previously reported in pure culture microorganisms, and subsequently toluene is degraded using a canonical pathway like that observed in other anaerobic microbes. The utilization of *hbs* instead of *bss* for fumarate addition to toluene after methylation of benzene suggested that Hbs may both methylate benzene to toluene and catalyze fumarate addition to the newly formed toluene. This would represent a unique mechanism not only to anaerobic benzene oxidation, but other anaerobic aromatic hydrocarbon oxidation. By converging benzene, toluene, and benzoate degradation into a single pathway, *G. daltonii* conserves resources, allowing it to thrive in contaminated environments.

This study has further demonstrated the transitional regulator AbrB in *G. daltonii* to be responsible for the repression of genes related to anaerobic aromatic hydrocarbon oxidation. While the ability to anaerobically degrade aromatic hydrocarbons has provided *G. daltonii* with a unique niche in which it can thrive, this process is particularly energy-intensive compared to the non-aromatic compound degradation. The ability to quickly switch between anaerobic aromatic and non-aromatic degradation pathways potentially provides *G. daltonii* with a mechanism to rapidly adapt to dynamic groundwater conditions.

## SUPPLEMENTAL MATERIAL

Supplemental material aem.00856-24-s0001.pdf Supplemental methods, Tables S1 to S4, and Figures S1 to S9.

ASM does not own the copyrights to Supplemental Material that may be linked to, or accessed through, an article. The authors have granted ASM a non-exclusive, world-wide license to publish the Supplemental Material files. Please contact the corresponding author directly for reuse.
