# Supplementary material for: Anaerobic benzene oxidation in Geotalea daltonii involves activation by methylation and is regulated by the transition state regulator AbrB
Source: Appl Environ Microbiol. 2024 Sep 17;90(10):e00856-24. doi: 10.1128/aem.00856-24 (PMC11497800; doi:10.1128/aem.00856-24)
Supplement: Supplemental material — Supplemental methods, Tables S1 to S4, and Figures S1 to S9. [file aem.00856-24-s0001.pdf]

Supplemental Materials for

Anaerobic Benzene Oxidation in *Geotalea daltonii* Involves Activation by Methylation and is Regulated by the Transition State Regulator AbrB

James E. Bullows, Alison Kanak, Lawrence Shedrick, Christina Kiessling, Muktak Aklujkar, Joel Kostka, Kuk-Jeong Chin

This file includes:

1. Supplemental Materials and Methods
2. Tables S1 – S4
3. Figures S1 – S9
4. References used in Supplemental Materials

1. Supplemental Materials and Methods

Analysis of toluene formation from benzene via cell lysates

All procedures were performed in an anaerobic chamber (Coy Laboratory, Grass Lake, MI). *G. daltonii* cells were harvested from mid-log growth phase cultures grown on benzene as sole carbon source and fumarate or nitrate as electron acceptor. Cell pellet was resuspended in 200 µL of lysis buffer [50 mM HEPES (pH 7.4), 150 mM NaCl, 10% glycerol, 1 mM EDTA, 1 mM benzamidine, and 1 mM DTT] and bead beaten for 1 min at 2,500 rpm via a Biospec Mini-BeadBeater-16 (Biospec Products, Bartlesville, OK). The resulting lysate was then centrifuged at 13,000 x g for 5 min. Supernatant was collected and analyzed for enzymatic activity as follows: 0.1 mL of supernatant was added to the analysis buffer [9.8 mL anaerobic lysis buffer with 1 mM of benzene] and gently vortexed. 0.1 mL samples were taken every 2 min for 20 min and aromatic metabolites were extracted using ethyl acetate. Metabolite concentration in aqueous phase was analyzed via HPLC as described in Materials and methods, *Analysis of organic substrates* section. 1 mM of fumarate or nitrate was added after 10 min. Reactions were halted after 20 min by exposure to oxygen.

Sequence overlap PCR (SO-PCR) for determination of operonic organization

To determine the arrangement of the *rpoN* operon, a method named sequence-overlap PCR (SO-PCR) was designed to map the genes as they are transcribed onto a single polycistronic mRNA strand (Fig. S5). Reverse primers RpoN_SO_1_R to RpoN_SO_6_R were designed to bind at 700 bp increments to the *lptC*, *lptA*, *lptB*, and *rpoN* genes. Six reverse transcription reactions were performed using primers RpoN_SO_1_R to RpoN_SO_6_R, respectively, and *G. daltonii* total RNA. Forward primers RpoN_SO_1_F to RpoN_SO_6_F were designed to bind within each overlapping region and were then used for PCR amplification, along with corresponding reverse primers and the previously elongated cDNA. PCR products were visualized by an agarose gel electrophoresis (Fig. S7) and sequenced via Sanger sequencing. The sequences were then concatenated via each overlapping segment, revealing a map of the operon, and confirming the arrangement of *rpoN* on an operon with *lptCAB*. One set of primers was designed as a negative control (RpoN_SO_6_F and RpoN_SO_6_R) to bind downstream of the *rpoN* gene onto Geob_2276 (annotated to encode for a HAD-IIIA family hydrolase), which was predicted by DOOR to be encoded on a different mRNA strand. A series of 5′ RACE procedures using the gene specific reverse primers were performed to determine the presence of any intra-operonic promoter regions.

DNA-protein binding analysis via electrophoretic mobility shift assay (EMSA)

The σ^{70} binding was tested by incubating pure σ^{70} saturated *E. coli* RNA polymerase holoenzyme (New England Biolabs, Ipswitch, MA) with a 511 bp amplicon covering the promoter region of the *rpoN* operon, as well as a 200 bp amplicon of *lptC* before performing an electrophoretic mobility shift assay (EMSA) (Fig. 8A). Various concentrations of σ^{70} ranging from 0.01-5.00 pmol were incubated with 1 pmol of the *rpoN* promoter region, 1 pmol of the σ^{54} dependent promoter *bss* operon, and a 150 bp segment of the housekeeping gene *recA* (included

in the incubation process to eliminate any nonspecific binding reactions between σ^{70} and the *rpoN* operon promoter), respectively.

To test AbrB binding, a 511 bp region upstream of *lptC* covering the entire promoter region of the *rpoN* operon was PCR amplified. Increasing concentrations of AbrB from 25-200 pmol were incubated with 1 pmol of the *rpoN* promoter region, the non-affiliated *raiA* promoter, and a 150 bp segment of the housekeeping gene *recA* (to eliminate any nonspecific binding reactions between AbrB and the *rpoN* operon promoter), respectively.

In vitro **expression analysis of AbrB**

G. daltonii cells were harvested from cultures grown to mid-log phase on benzene, toluene, and acetate, respectively, before being pelleted inside of the anaerobic serum bottle for 10 min at 4,300 x g, 4°C. Growth media was removed from serum bottles anaerobically by syringe, leaving only pelleted cells. Pellet was washed twice with 50 mL of anaerobic electroporation buffer. The buffer was removed anaerobically via syringe after centrifugation. Cell pellet was then resuspended in electroporation buffer to 10^{11} cells/mL. A prepared solution of DMSO: electroporation buffer (60:40 [vol:vol]) was added to cells to 10% vol/vol. 25 µL of prepared cells were combined with 2 µL of the expression plasmid pETDuet-1 carrying an IPTG-inducible *abrB* gene (pETDuet-1-*abrB*) within a nitrogen-purged electroporation cuvette and electroporated 25 kV/cm for 100 µs. Resulting transformants were transferred via anaerobic syringe to a fresh anerobic serum bottle containing 100 mL of freshwater media. Cells were incubated for 5 hours at 30°C before the addition of appropriate antibiotic. Protein expression was induced via addition of anaerobic IPTG to 1 mM final concentration under the following conditions: with IPTG added on the day of transformation; without the addition of IPTG over the life of the culture; and with IPTG added during mid-log growth. Transformants growing on aromatic compounds also included IPTG addition during mid-log growth, followed by acetate addition during death phase. Each condition was performed in triplicate.

2. Supplemental Tables

Table S1. Primers used in this study*

Primer	Target	Sequence	Amplicon length (bps)
BssAGD-F	*bssA*	5'-ACTTCGCATCGATCACGTCCA-3'	119
BssAGD-R		5'-ACCTGGCGCTGAATCCTGA-3'	
HbsA-F	*hbsA*	5'-ACTTGCTCAATCAGCGGCTC-3'	133
HbsA-R		5'-TCAGCGGTACTCACGCAGT-3'	
BbsF-F	*bbsF*	5'-CCTTCAAGGATGGGATCAAC-3'	240
BbsF-R		5'-TCATGGTAACGTAGATGATCCG-3'	
BbsA-F	*bbsA*	5'-TCAGGAAGAAGCCCAGAA-3'	176
BbsA-R		5'-GGAGAGAGGGATTATCTCGAA-3'	
BamA-F	*bamA*	5'-AGTTGGCGACAAGGCATTCTGT-3'	317
BamA-R		5'-TTGCTTGTTCGCAGCCGATCAT-3'	
BamR-F	*bamR*	5'-TTGTGCCCAGATGCTGAAGA-3'	256
BamR-R		5'-GGAAAACAGCAGGTCTTCAG-3'	
PpcB-F	*ppcB*	5'-ATCAACCGCACGGCAACATT-3'	122
PpcB-R		5'-ATCGCCTCGGTAAAGCAGAT-3'	
0241-F	Methyltransferase gene Geob_0241	5'-CCATTACGGACATAACGACCTTG-3'	166
0241-R		5'-CCTGTCAAGCTTGATATCCATTGC-3'	
AbrB-NcoI-F	Whole *abrB* gene, including NcoI restriction site	5'-CCATGGGAAGCGGTCAA-3'	251
AbrB-AvrII-R	Whole *abrB* gene, including AvrII restriction site	5'-CCTAGGCGAGATATTCGAGCCA-3'	
RpoN-P-F	*rpoN* Promoter Region	5'-GAGCTCGCTATCGGCATCGT-3'	511
RpoN-P-R		5'-ACACCGGCCAGAACTATGG-3'	
AbrBGD-F	*abrB* gene	5'-CCAACGGATGCAGGTAATCG-3'	127
AbrBGD-R		5'-TCATAAGCGGTCCGATTCCC-3'	
RpoNGD-F	*rpoN* gene	5'-AGTAGCCACTGTCTGCGGAA-3'	152
RpoNGD-R		5'-CGACCAGACTCCCACCAATAC-3'	
BenzCoAT_F	Benzoate CoA transferase gene Geob_2194	5'-CGGGTAAATGCCTGGTTCAC-3'	122
BenzCoAT_R		5'-TCGATAGGCCAGAATTCCCG-3'	
RpoN_SO_1_F	*rpoN* Sequence Overlap Segment 1	5'-CGGCACCGTTATTACAAATGAG-3'	205
RpoN_SO_1_R		5'-CATGGCAAGGATAAGCCTGATC-3'	
RpoN_SO_2_F	*rpoN* Sequence Overlap Segment 2	5'-CGGTAAATTATGCTGAGACCGAA-3'	573
RpoN_SO_2_R		5'-CACTTGCTGTGCCCGTTCAT-3'	
RpoN_SO_3_F	*rpoN* Sequence Overlap Segment 3	5'-AGCGGTGTCGGCATGGAACTT-3'	793
RpoN_SO_3_R		5'-GGCACAGACCAACCATCATGTA-3'	
RpoN_SO_4_F	*rpoN* Sequence Overlap Segment 4	5'-TTGCATAGCAGTGACCTGAA-3'	861
RpoN_SO_4_R		5'-GAAGGAGCTTTATTGCCTGTTG-3'	
RpoN_SO_5_F	*rpoN* Sequence Overlap Segment 5	5'-TCCCTCAGTGCAGAGGTGTA-3'	717
RpoN_SO_5_R		5'-CCGAGATGTACTGGACATCGT-3'	
RpoN_SO_6_F	*rpoN* Sequence Overlap Segment 6	5'-CCATCATCTCAAGGATCTGGAA-3'	883
RpoN_SO_6_R		5'-ATGTGCCTGAATGTCGTGGTAA-3'	
RaceUT	Poly C tail of 5′ RACE product	5'-CGCGAATTCCTCTTCTAGATGGGIIGGGIIGGGIIG-3'	NA
RaceU	RaceUT primer	5'-CGCGAATTCCTCTTCTAGATGG-3'	NA
LptC_R	Upstream of the *rpoN* promoter region	5'-CTGCAGTGCCATCTCCACAT-3'	NA
LptC_R_Nest	Inside of the LptC_R primer product	5'-CATGGCAAGGATAAGCCTGATC-3'	NA
Bss1RaceL	Inside of *bssD*	5'-TGTGGCACCATGGGCAATGGAGTGGAC-3'	NA

*All primers listed were designed in this study.

Table S2. Calculated electron balances for *G. daltonii* grown on benzene as a sole carbon source and electron donor and fumarate or nitrate as electron acceptor

A. Culture grown on benzene and fumarate

Electron equivalents calculated from consumed benzene (µM)	Electron equivalents recovered in succinate measured (µM)	Calculated electron recovery (%)
**3,285**	**3,982**	**121.2 ± 23**

Calculated electron recovery is shown as the percentage of electron equivalents recovered in succinate production assuming complete oxidation of benzene to CO_2.

B. Culture grown on benzene and nitrate

Electron equivalents calculated from consumed benzene (µM)	Electron equivalents recovered in nitrate loss measured (µM)	Calculated electron recovery (%)
**792**	**860**	**108.6 ± 17**

Calculated electron recovery is shown as the percentage of electron equivalents recovered in nitrate loss assuming complete oxidation of benzene to CO_2.

Table S3. Enzymes expressed in the toluene degradation pathway but not in the benzoate degradation pathway in *G. daltonii***.** There are 9 enzymes expressed during toluene degradation but not during benzoate degradation.

**Enzymes Unique to Toluene Degradation**	**Protein Size**
Benzylsuccinate synthase (BssA) alpha subunit	95 kDa
Benzylsuccinate synthase (BssB) beta subunit	8 kDa
Benzylsuccinate synthase (BssC) gamma subunit	6 kDa
Succinyl-CoA:benzylsuccinate CoA-transferase (BbsE) subunit	45 kDa
Succinyl-CoA:benzylsuccinate CoA-transferase (BbsF) subunit	45 kDa
(R)-benzylsuccinyl-CoA dehydrogenase (BbsG)	45 kDa
E-phenylitaconyl-CoA hydratase (BbsH)	28 kDa
2-[hydroxy(phenyl)methyl]-succinyl-CoA dehydrogenase (BbsC) subunit	27 kDa
2-[hydroxy(phenyl)methyl]-succinyl-CoA dehydrogenase (BbsD) subunit	27 kDa

Table S4. Genes encoding for proteins predicted to be involved in the degradation of aromatic compounds in *G. daltonii*

Gene ID	Gene Annotation	Gene Name
Geob_0103	4-hydroxybenzoyl-CoA reductase molybdenum cofactor biosynthesis protein	*pcmW*
Geob_0104	4-hydroxybenzoyl-CoA reductase/selenium-dependent molybdenum hydroxylase system protein, YqeB family	*pcmV*
Geob_0105	4-hydroxybenzoyl-CoA reductase (molybdopterin cytosine dinucleotide)-dioxomolybdenum--sulfide ligation chaperone	*pcmU*
Geob_0106	4-hydroxybenzoyl-CoA reductase, alpha subunit	*pcmT*
Geob_0107	4-hydroxybenzoyl-CoA reductase, iron-sulfur cluster-binding subunit	*pcmS*
Geob_0108	4-hydroxybenzoyl-CoA reductase, flavoprotein subunit	*pcmR*
Geob_0110	Twin arginine translocase protein A	*tatA*
Geob_0114	Short-chain dehydrogenase/reductase SDR	*pcmM*
Geob_0117	P-cresol transport outer membrane protein	*pcmK*
Geob_0118	P-cresol methylhydroxylase, alpha subunit	*pcmJ*
Geob_0119	4-cresol dehydrogenase (hydroxylating) flavoprotein subunit	*pcmI*
Geob_0120	Twin-arginine translocation pathway signal	*pcmH*
Geob_0121	P-cresol methylhydroxylase, beta subunit cytochrome c, 1 heme-binding site	*pcmG*
Geob_0122	Menaquinol oxidoreductase complex, cytochrome c subunit, 4 heme-binding sites	*pcmF*
Geob_0123	Menaquinol oxidoreductase complex, iron-sulfur cluster-binding subunit	*pcmE*
Geob_0124	Menaquinol oxidoreductase complex, membrane protein subunit	*pcmD*
Geob_0125	Menaquinol oxidoreductase complex, cytochrome b subunit	*pcmC*
Geob_0126	FAD-binding pyridine nucleotide-disulfide oxidoreductase	*pcmB*
Geob_0138	CBS domain containing protein (binds ATP, makes enzymes sensitive to ATP)	*ppsC*
Geob_0140	Phenylphosphate carboxylase, beta subunit	*ppcB*
Geob_0141	Pyruvate phosphate dikinase PEP	*ppsB*
Geob_0142	PEP-utilising protein	*ppsA*
Geob_0144	Histidine kinase	*bamV*
Geob_0145	Response regulator	*bamW*
Geob_0193	Aromatic acid transporter	*benK*
Geob_0200	Benzoate-coenzyme A ligase	*bamY*
Geob_0212	Benzoyl-CoA reductase, bis-(molybdopterin)-oxotungsten-binding subunit	*bamB-1*
Geob_0213	Benzoyl-CoA reductase, iron-sulfur cluster-binding subunit	*bamC-1*
Geob_0214	Iron-sulfur cluster-binding oxidoreductase, CCG domain pair-containing, putative benzoyl-CoA reductase electron transfer protein	*bamD-1*
Geob_0215	Polyferredoxin, putative benzoyl-CoA reductase electron transfer protein	*bamE-1*
Geob_0216	Benzoyl-CoA reductase electron transfer protein, selenocysteine-containing, putative	*bamF-1*
Geob_0217	Benzoyl-CoA reductase electron transfer protein, putative	*bamG-1*
Geob_0218	Benzoyl-CoA reductase electron transfer protein, putative	*bamH-1*
Geob_0219	Iron-sulfur cluster-binding protein, putative	*bamI-1*
Geob_0227	Benzoyl-CoA reductase, bis-(molybdopterin)-oxotungsten-binding subunit	*bamB-3*
Geob_0228	Benzoyl-CoA reductase, bis-(molybdopterin)-oxotungsten-binding subunit	*bamB-4*
Geob_0229	Benzoyl-CoA reductase, iron-sulfur cluster-binding subunit	*bamC-3*
Geob_0230	Iron-sulfur cluster-binding oxidoreductase, CCG domain pair-containing, putative benzoyl-CoA reductase electron transfer protein	*bamD-2*
Geob_0231	Polyferredoxin, putative benzoyl-CoA reductase electron transfer protein	*bamE-2*
Geob_0232	Benzoyl-CoA reductase electron transfer protein, selenocysteine-containing	*bamF-2*
Geob_0233	Benzoyl-CoA reductase electron transfer protein, putative	*bamG-2*
Geob_0234	Benzoyl-CoA reductase electron transfer protein, putative	*bamH-2*
Geob_0235	Iron-sulfur cluster-binding protein	*bamI-2*
Geob_0095	Electron transfer flavoprotein subunit beta	*bamO*
Geob_0096	Electron transfer flavoprotein subunit alpha	*bamP*
Geob_0097	6-hydroxycyclohex-1-ene-1-carbonyl-CoA dehydrogenase	*bamQ*
Geob_0098	Cyclohexa-1,5-dienecarbonyl-CoA hydratase	*bamR*
Geob_0100	Acetyl-CoA acetyltransferase	*bamN*
Geob_0101	Metal-dependent hydrolase, putative	*bamU*
Geob_2097	Outer membrane protein assembly factor BamA	*bamA*
Geob_0622	Outer membrane protein assembly factor BamA	*bamA*
Geob_2448	Benzylsuccinate synthase alpha subunit	*bssA*
Geob_2448	Benzylsuccinate synthase beta subunit	*bssB*
Geob_2449	Benzylsuccinate synthase gamma subunit	*bssC*
Geob_2450	Benzylsuccinate synthase activase	*bssD*
Geob_2427	Succinyl-CoA:benzylsuccinate CoA transferase A subunit	*bbsA*
Geob_2428	Succinyl-CoA:benzylsuccinate CoA transferase B subunit	*bbsB*
Geob_2429	2-[hydroxy(phenyl)methyl]-succinyl-CoA dehydrogenase C subunit	*bbsC*
Geob_2430	2-[hydroxy(phenyl)methyl]-succinyl-CoA dehydrogenase D subunit	*bbsD*
Geob_2421	Benzylsuccinate CoA-transferase E subunit	*bbsE*
Geob_2422	Benzylsuccinate CoA-transferase F subunit	*bbsF*
Geob_2423	(R)-benzylsuccinyl-CoA dehydrogenase	*bbsG*
Geob_2424	E-phenylitaconyl-CoA hydratase	*bbsH*
Geob_2437	3-hydroxybenzylsuccinate synthase beta subunit	*hbsB*
Geob_2438	3-hydroxybenzylsuccinate synthase alpha subunit	*hbsA*
Geob_2439	3-hydroxybenzylsuccinate synthase gamma subunit	*hbsC*
Geob_2440	3-hydroxybenzylsuccinate synthase activase	*hbsD*
Geob_2441	Sigma-54-dependent Fis family transcriptional regulator	*hbsR*
Geob_2451	Sigma-54-dependent Fis family transcriptional regulator	*bssR*

3. Supplemental Figures

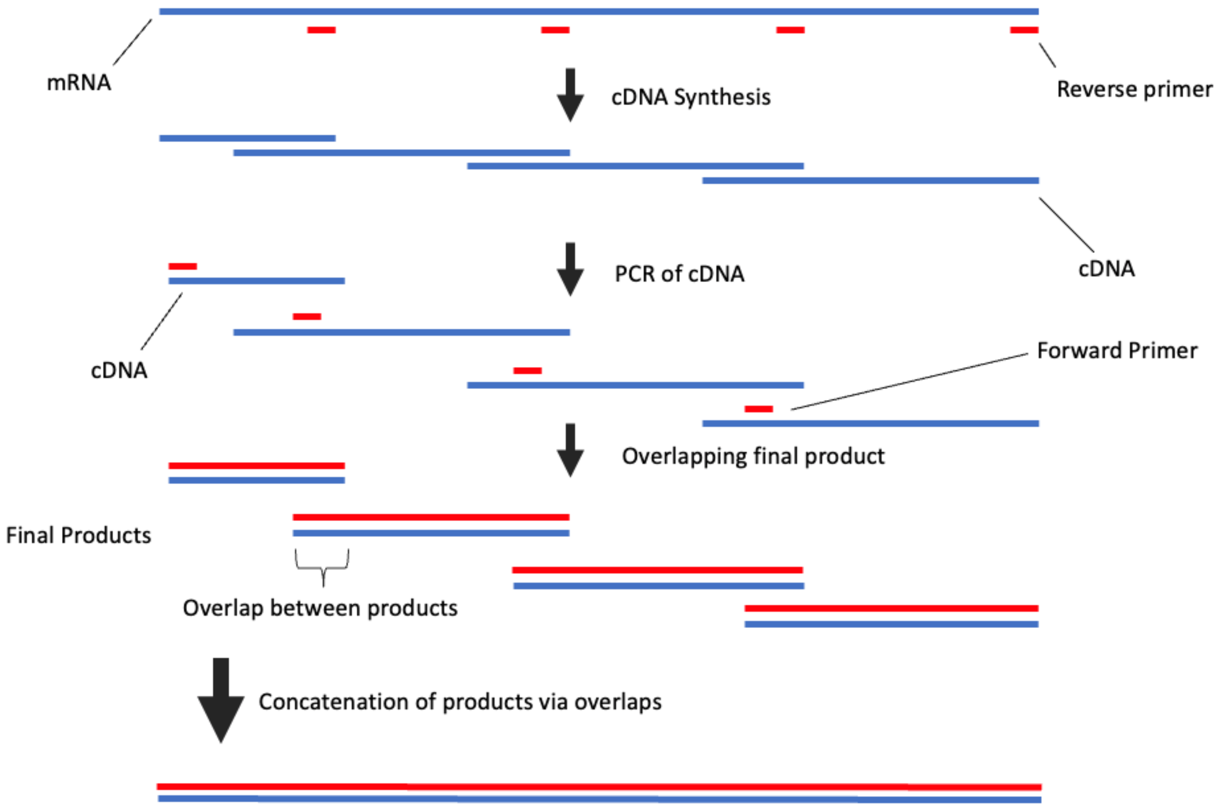

Figure S1. Sequence overlap PCR method. Total mRNA extract is subjected to RT-PCR using a series of reverse primers designed to bind at 700 bp intervals over the proposed operon. Each cDNA product will overlap with the neighboring cDNA synthesized via the preceding and succeeding reverse primers. These amplicons were sequenced, and the sequences were concatenated using the overlapping regions. If the genes are not encoded onto a single operon, truncated PCR products without overlapping regions will be produced and concatenation will not be possible.

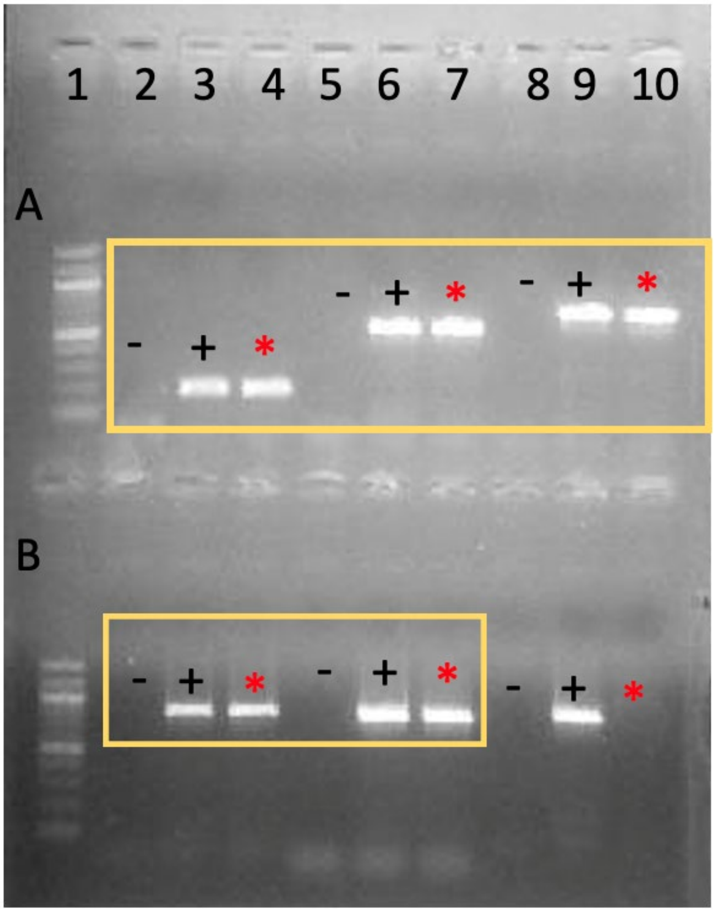

Figure S2. Products of sequence overlap RT-PCR. Each product represents a portion of the target operon. *Lane A1:* 100 bp DNA ladder. *Lane A2:* Negative control product of RponN_SO_1. *Lane A3:* Positive control product of primers RpoN_SO_1. *Lane A4:* Product of RT-PCR of primers RpoN_SO_1 from total *G. daltonii* RNA. *Lane A5:* Negative control product of primers RpoN_SO_2. *Lane A6:* Positive control product of primers RpoN_SO_2. *Lane A7:* Product of RT-PCR of primers RpoN_SO_2 from total *G. daltonii* RNA. *Lane A8:* Negative control product of primers RpoN_SO_3. *Lane A9:* Positive control product of primers RpoN_SO_3. *Lane A10:* Product of RT-PCR of primers RpoN_SO_3 from total *G. daltonii* RNA. *Lane B1:* 100 bp standard ladder. *Lane B2:* Negative control product of primers RpoN_SO_4. *Lane B3:* Positive control product of primers RpoN_SO_4. *Lane B4:* Product of RT-PCR of primers RpoN_SO_4 from total *G. daltonii* RNA. *Lane B5:* Negative control product of primers RpoN_SO_5. *Lane B6:* Positive control product of primers RpoN_SO_5. *Lane B7:* Product of RT-PCR of primers RpoN_SO_5 from total *G. daltonii* RNA. *Lane B8:* Negative control product of primers RpoN_SO_6. *Lane B9:* Positive control product of primers RpoN_SO_36 *Lane B10:* Product of RT-PCR of primers RpoN_SO_6 from total *G. daltonii* RNA; reverse primer was deliberately designed upstream of the target operon as a negative control.

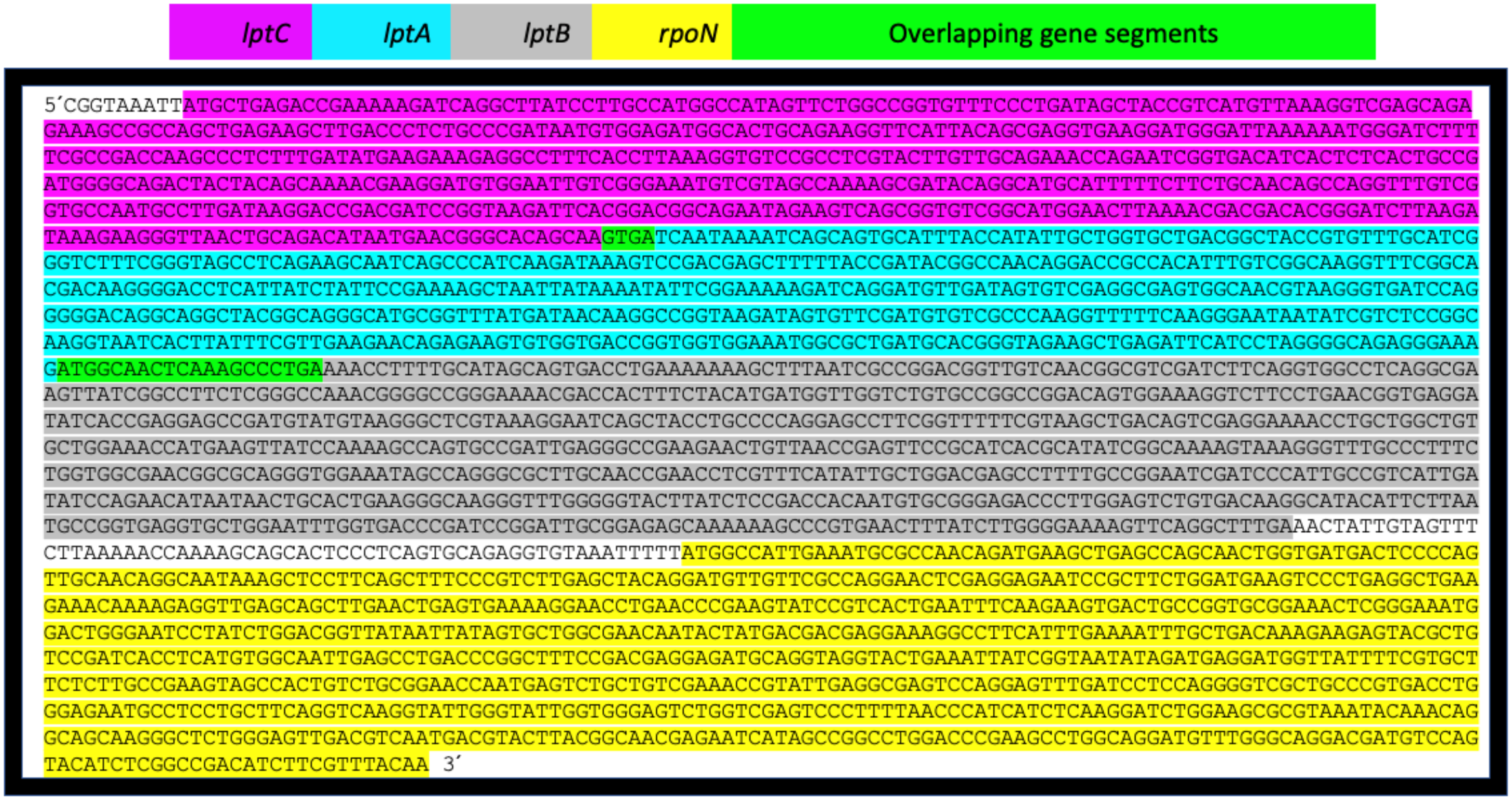

Figure S3. Sequence overlap PCR arrangement. After the concatenation of the various SO-PCR products, a single mRNA sequence was determined bioinformatically showing the arrangement of the *lptCAB* genes with the *rpoN* gene.

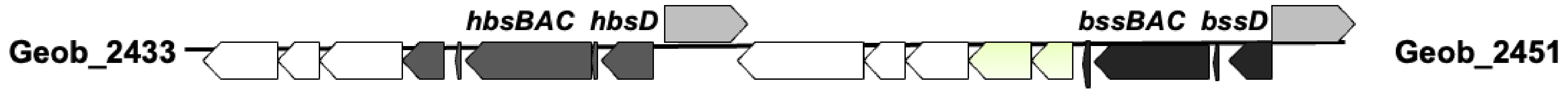

Figure S4. The *hbs* and *bss* gene clusters in *G. daltonii*. Organization of anaerobic toluene oxidation genes within the genome of *G. daltonii*. White arrows indicate genes without predicted aromatic compound degradation functions according to NCBI annotations. Dark grey arrows indicate genes predicted to be responsible for Bss formation and activation. Light grey arrows indicate similar predicted sigma 54-dependent transcriptional regulators. Spaces within arrows indicate predicted non-coding regions between genes and/or operons.

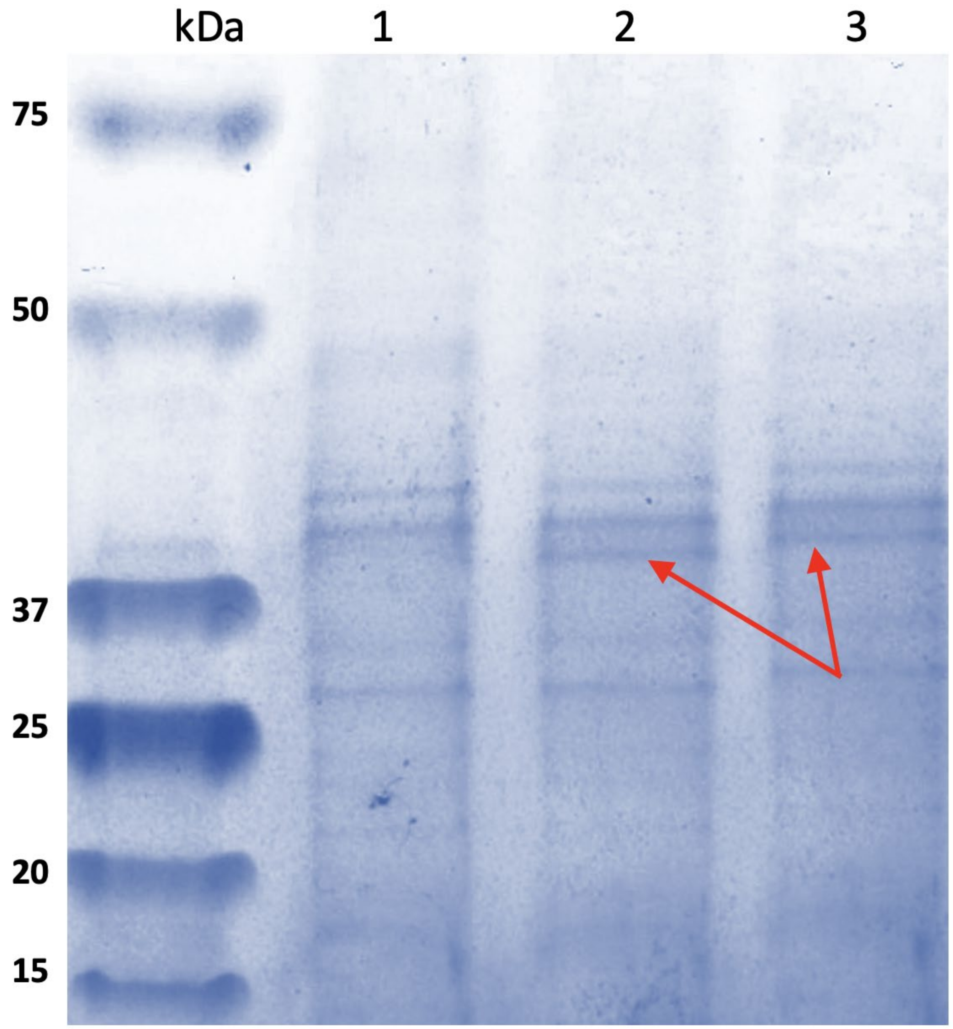

Figure S5. SDS-PAGE image showing the protein profiles of *G. daltonii* whole cell lyates. Lane 1: cells grown on benzoate as sole carbon source; lane 2: cells grown on benzene as sole carbon source; lane 3: cells grown on toluene as sole carbon source. Arrows indicate the location of succinyl-CoA:benzylsuccinate CoA-transferase subunits BbsE and BbsF, and (R)-benzylsuccinyl-CoA dehydrogenase, *ca*. 45 kDa.

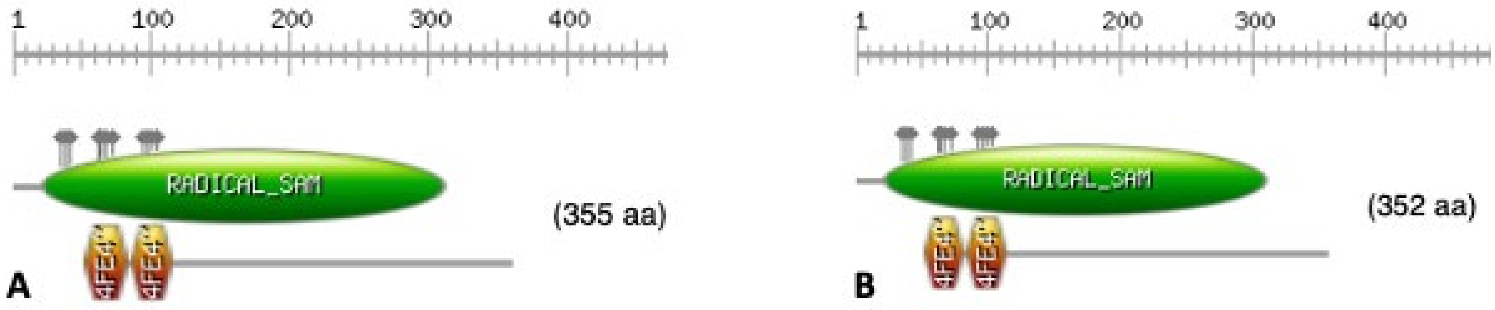

Figure S6. Functional SAM sites of Hbs and Bss Activase. Diagrams of protein functional sites and domains (1) identified in (A) Hbs D and (B) Bss D Both proteins contain radical SAM core domain profiles and two 4Fe-4S ferredoxin-type iron-sulfur binding domain profiles.

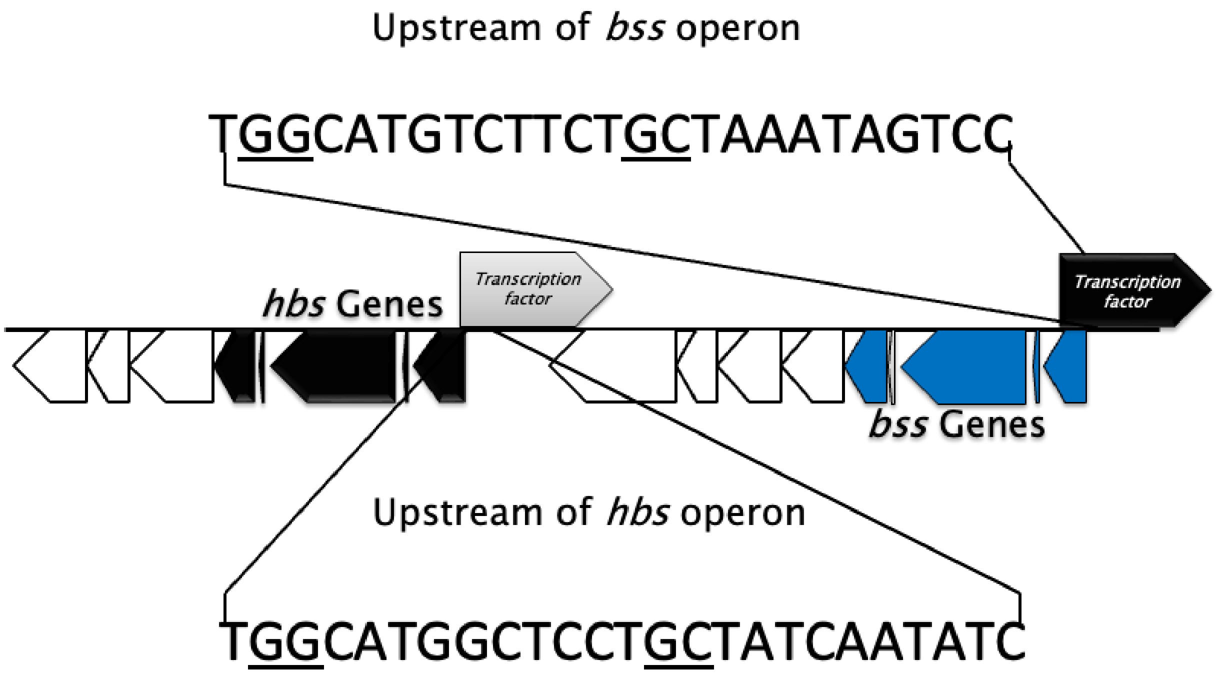

Figure S7. Sigma 54-like Promoters in *G. daltonii.* Diagram showing coding and upstream regions of *bss* (Geob_2442-Geob_2450) and *hbs* (Geob_2431-Geob_2441) genes in *G. daltonii.* Black and blue arrows represent *hbs* and *bss* genes, respectively. Transcription factors are located downstream and in opposite directions of the modulated genes.

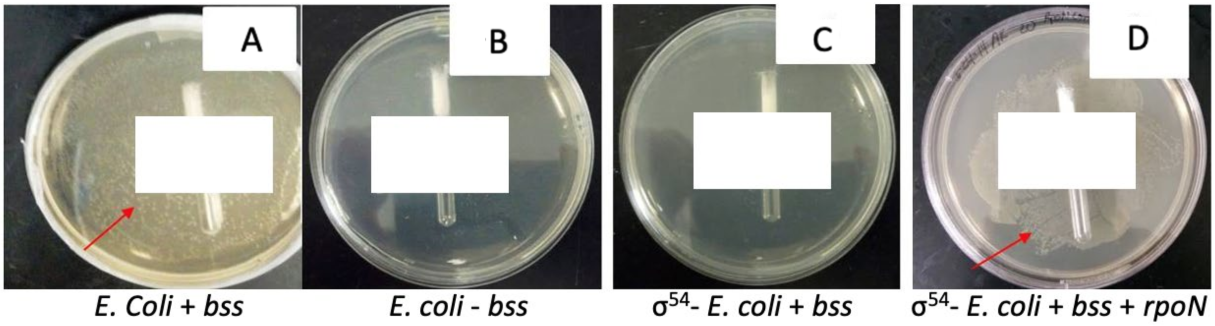

Figure S8. Sigma 54 is required for transcription of *bss* genes in *E. coli*. A. *E. coli* transformed with *bss* operon. B. Untransformed *E. coli*. C. σ^{54}- (deficient) *E. coli* transformed with plasmid containing *G. daltonii bss* operon. D. σ^{54}- *E. coli* transformed with plasmid containing *G. daltonii bss* operon and *G. daltonii rpoN* gene. Red arrows indicate colony growth.

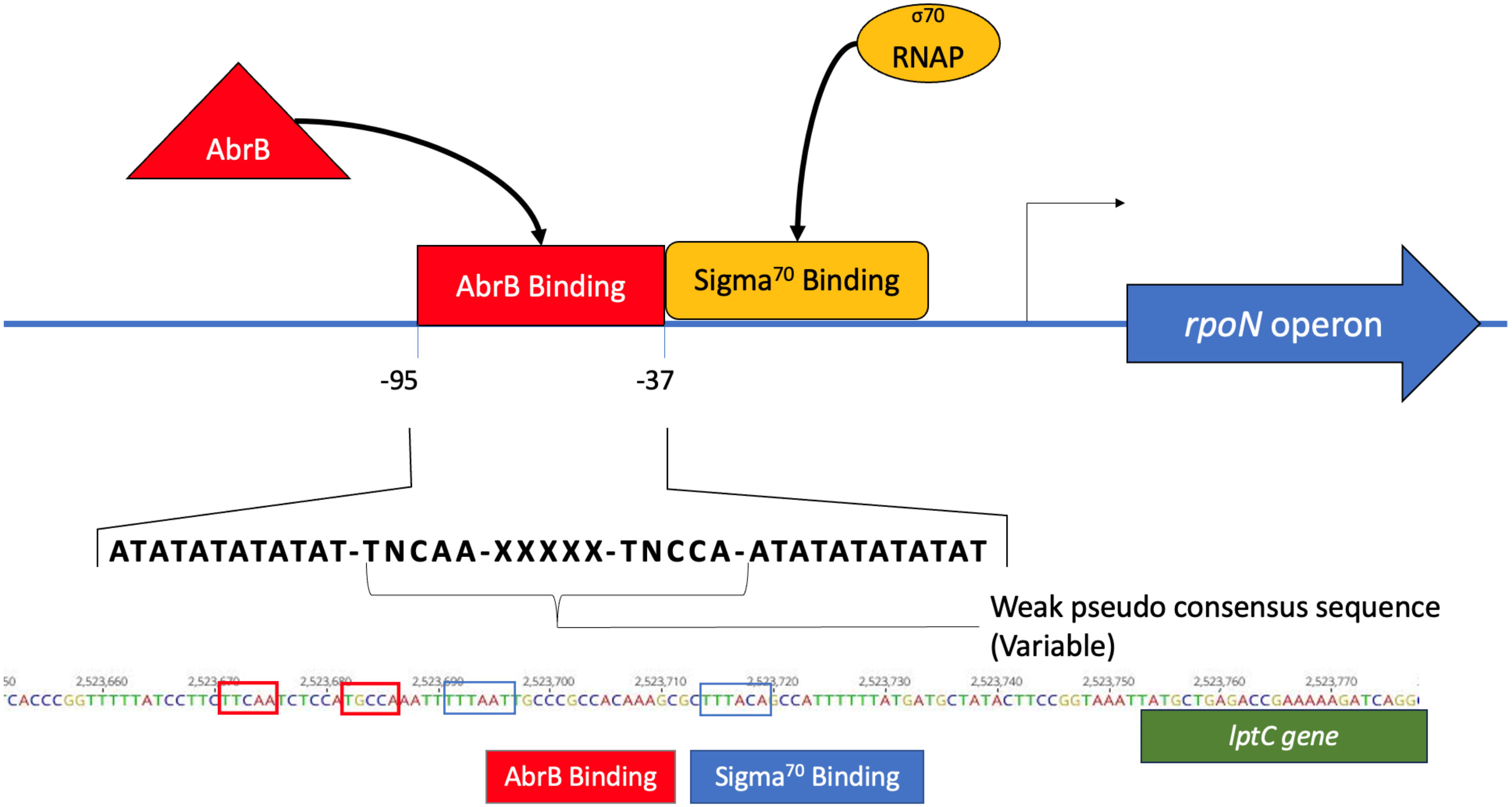

Figure S9. The *rpoN* operon is proceeded by σ^{70} and AbrB binding sites in the promoter region**.** 5′ RACE revealed binding sites for AbrB and σ^{70} upstream of the *rpoN* operon within the promoter region.

4. References used in Supplemental Materials

1. de Castro E, Sigrist CJ, Gattiker A, Bulliard V, Langendijk-Genevaux PS, Gasteiger E, Bairoch A, Hulo N. 2006. ScanProsite: detection of PROSITE signature matches and ProRule-associated functional and structural residues in proteins. Nucleic Acids Res 34:W362-W365.
